# Vitamin B5 and succinyl-CoA improve ineffective erythropoiesis in *SF3B1* mutated myelodysplasia

**DOI:** 10.1126/scitranslmed.abn5135

**Published:** 2023-03-01

**Authors:** Syed A Mian, Céline Philippe, Eleni Maniati, Pantelitsa Protopapa, Tiffany Bergot, Marion Piganeau, Travis Nemkov, Doriana Di Bella, Valle Morales, Andrew J Finch, Angelo D’Alessandro, Katiuscia Bianchi, Jun Wang, Paolo Gallipoli, Shahram Kordasti, Anne Sophie Kubasch, Michael Cross, Uwe Platzbecker, Daniel H Wiseman, Dominique Bonnet, Delphine G Bernard, John G Gribben, Kevin Rouault-Pierre

**Affiliations:** 1 The Francis Crick Institute, London NW1 1AT, United Kingdom; 2 Barts Cancer Institute, Queen Mary University of London, London EC1M 6BQ, United Kingdom; 3 University of Brest, Inserm, EFS, UMR1078, GGB, 29238 Brest, France; 4 Department of Biochemistry and Molecular Genetics, University of Colorado Denver - Anschutz Medical Campus, Aurora, CO 80045, USA; 5 System Cancer Immunology, Comprehensive Cancer Centre, King's College London, London WC2R 2LS, United Kingdom; 6 Department of Hematology, Cellular Therapy and Hemostaseology, Leipzig University Hospital, 04103 Leipzig, Germany; 7 Division of Cancer Sciences, The University of Manchester, Manchester M20 4GJ, UK; 8 Centre de Ressources Biologiques du CHRU de Brest, 29238 Brest, France

## Abstract

Patients with myelodysplastic syndrome and ring sideroblasts (MDS-RS) present with symptomatic anemia due to ineffective erythropoiesis that impede their quality of life and increase morbidity. More than 80% of patients with MDS-RS harbor splicing factor 3B subunit 1 (SF3B1) mutations, the founder aberration driving MDS-RS disease. Here, we report how mis-splicing of coenzyme A synthase (*COASY*), induced by mutations in *SF3B1*, impacts heme biosynthesis and erythropoiesis. Our data revealed that *COASY* was upregulated during normal erythroid differentiation, and its silencing prevented the formation of erythroid colonies, impeded erythroid differentiation, and precluded heme accumulation. In patients with MDS-RS, loss of protein due to *COASY* mis-splicing led to depletion of both CoA and succinyl-CoA. Notably, supplementation with COASY substrate (vitamin B5) rescued CoA and succinyl-CoA concentrations in *SF3B1*^mut^ cells and mended erythropoiesis differentiation defects in MDS-RS primary patient cells. Our findings reveal a key role of the coenzyme A synthesis pathway in erythroid maturation and identify upstream as well as downstream metabolites of COASY as a potential treatment for anemia in patients with MDS-RS.

## Introduction

Myelodysplastic syndrome (MDS) is a clonal stem cell disorder that increases markedly with age and has a high propensity to progress to acute myeloid leukemia (AML)(1, 2). Ineffective erythropoiesis and anemia are hallmarks of MDS (3). In low-risk MDS without del5q, (revised International Prognostic Scoring System for MDS risk: very low/low/intermediate), erythropoiesis stimulating agents that stimulate red blood cell production are the first line of treatment (2), with an initial response rate of approximately 50%. However responders to treatment will eventually become resistant, with a median response duration of 18 to 24 months(4–8), further reduced in patients with myelodysplastic syndrome and ring sideroblasts (MDS-RS) (7). The addition of hypomethylating agents or lenalidomide to the treatment regimen do not improve these patients’ overall survival (9). Hence, most patients eventually become dependent on red blood cell transfusions, which contribute to iron overload associated with reduced quality of life and an increased risk of progression to AML (10). The failure of erythropoiesis stimulating agents mainly limits second line therapeutic options to luspatercept, a transforming growth factor-β superfamily ligand trap. This agent promotes late-stage erythropoiesis and leads to transfusion independency (11) in up to 38% of patients with MDS-RS (NCT02631070) (12, 13). The incidence of splicing factor mutation is high in general in MDS (14), making these patients ideal candidates for spliceosome inhibitors such as H3B-8800 or E7107. However, recent studies have shown low response rates and major toxicities in patients who received these inhibitors (15). Given that MDS is an age-related disease with an indolent clinical course and that our general populations are aging, MDS cases cannot but increase in the future. Therefore, there is clearly an unmet need to develop therapeutic approaches to treat this disease.

**Figure 1:**
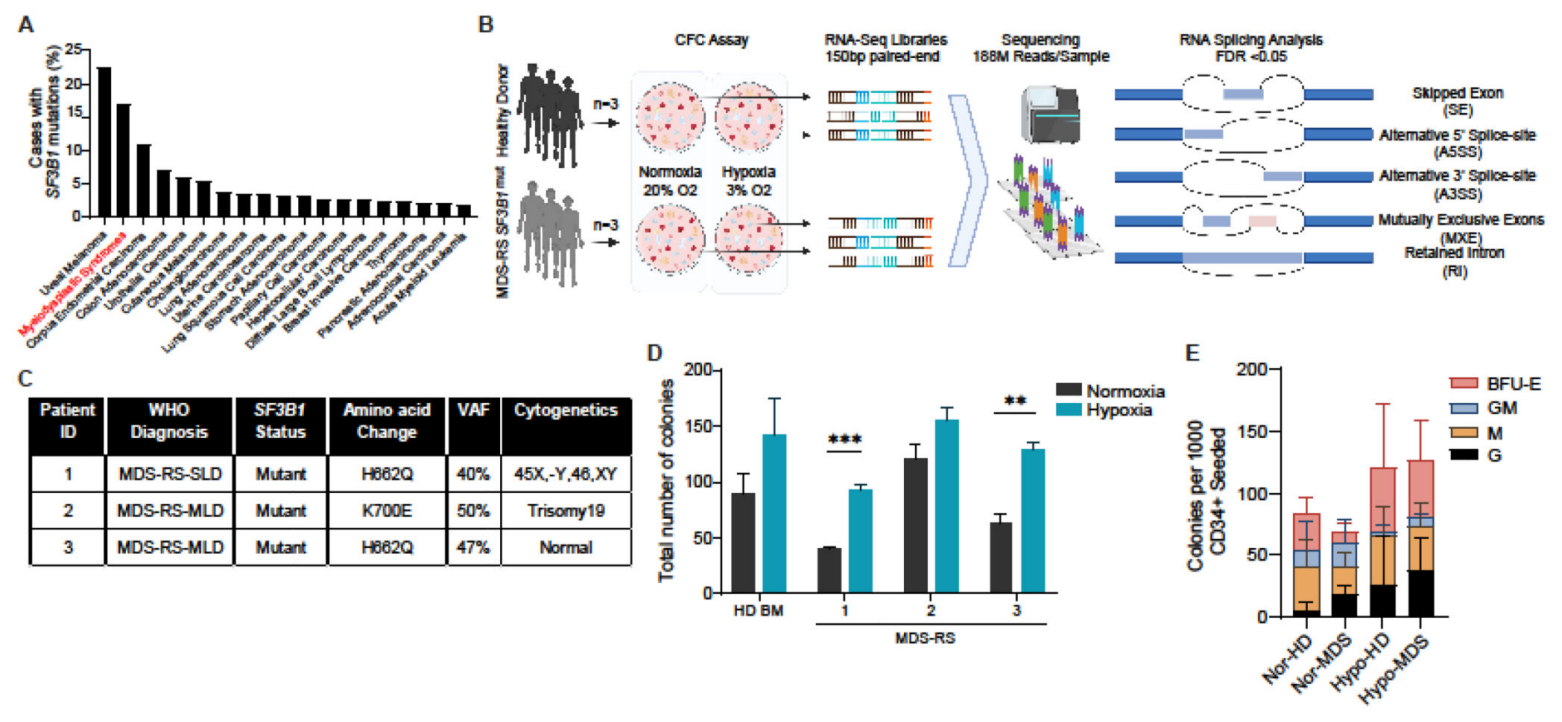
RNA splicing analysis is performed on *SF3B1^mut^* haematopoietic progenitors derived from colony forming assay. **(A)** Presence of *SF3B1* mutations across cancers in TCGA and MSKCC (MDS) 2020 cohorts. **(B)** Schematic of the experimental strategy used to identify mis-splicing events associated with *SF3B1* mutations. BM CD34^+^ cells from healthy donors (HD1, HD2, HD3) and 3 patients with MDS *SF3B1*^mut^ were seeded in methylcellulose to generate colonies under hypoxic or normoxic conditions. Colonies from each condition were harvested and used for RNA-sequencing. Splicing analysis with rMATS identified splicing events with a FDR<0.05. Splicing events were classified into 5 subcategories: skipped exon (SE), alternative 5’ splice site (A5SS), alternative 3’ splice site (A3SS), mutually exclusive exon (MXE), and retained intron (RI). **(C)** Clinical and *SF3B1* mutations details of the 3 patients used for RNA sequencing analysis. **(D)** Total number of colonies derived from the BM CD34^+^ cells from healthy donors (n=3) and patients with MDS-RS (n=3) per 1000 CD34^+^ cells seeded. Colony forming cell (CFC) assays were performed under hypoxic (3% O_2_) or normoxic conditions (20% O_2_). Data are the mean± S.E.M. ** *p<0.01*, *** *p<0.001*. **(E)** Bar chart showing the number of BFU-E/G/M/GM colonies obtained from 3 healthy donors or 3 MDS-RS *SF3B1^mut^* patients’ samples per 1000 CD34^+^ cells plated and cultured upon normoxia or hypoxia (n=3).

*SF3B1* mutant low-risk MDS represents a distinct entity, mainly characterized by ineffective erythropoiesis and strongly associated with a ring sideroblast phenotype (16–19). In addition to being the most frequently mutated gene in MDS (14) and MDS-RS, *SF3B1* is amongst the most altered splicing factor across all cancers (20) (Fig 1A). All *SF3B1* hotspot mutations cluster in the C-terminal HEAT repeat domains (21), creating a neomorphic activity that induces mis-splicing through cryptic 3′ splice site selection (22) (22). Transcriptional profiling of *SF3B1* mutant cells has revealed widespread mRNA splicing alterations (23–26). Due to discrepancies in human and mice ribonucleic sequences, murine models of *SF3B1^mut^* have failed to recapitulate the common mis-splicing events identified in patient clinical samples (27). Therefore, working on primary human samples is critical in order to dissect the role of aberrant splicing in MDS pathogenesis. To identify alternative splice variants, RNA splicing analysis is usually performed using bone marrow (BM) CD34^+^ hematopoietic stem/progenitor cells (HSPCs) or mononuclear cells from patients with MDS (16, 18, 19, 28, 29). Because MDS-RS is characterized by differentiation block, we hypothesized that performing RNA splicing analysis on cells undergoing differentiation (colonies derived from patient HSPCs) would reveal key splicing events involved in MDS *SF3B1^mut^* biology.

Here, we identified a mis-splicing in the 5’UTR of the coenzyme A synthase (*COASY*) as a major contributor to ineffective erythropoiesis in patients with MDS-RS. In brief, we explored the consequences of *COASY’*s mis-splicing in *SF3B1^mut^* cells and used primary hematopoietic stem/progenitor cells to investigate the role of COASY during normal erythroid differentiation and heme synthesis. Lastly, we explored the therapeutic value of treating *ex-vivo* MDS-RS primary patient cells with COASY’ substrates and by-products. Our results suggest that targeting the COASY/CoA/Succinyl-CoA axis in MDS patients with *SF3B1* mutation could represent a highly relevant strategy to treat ineffective erythropoiesis.

## Results

### *SF3B1* mutations induce mis-splicing of *COASY* isoforms in patients with MDS-RS

**Figure 2:**
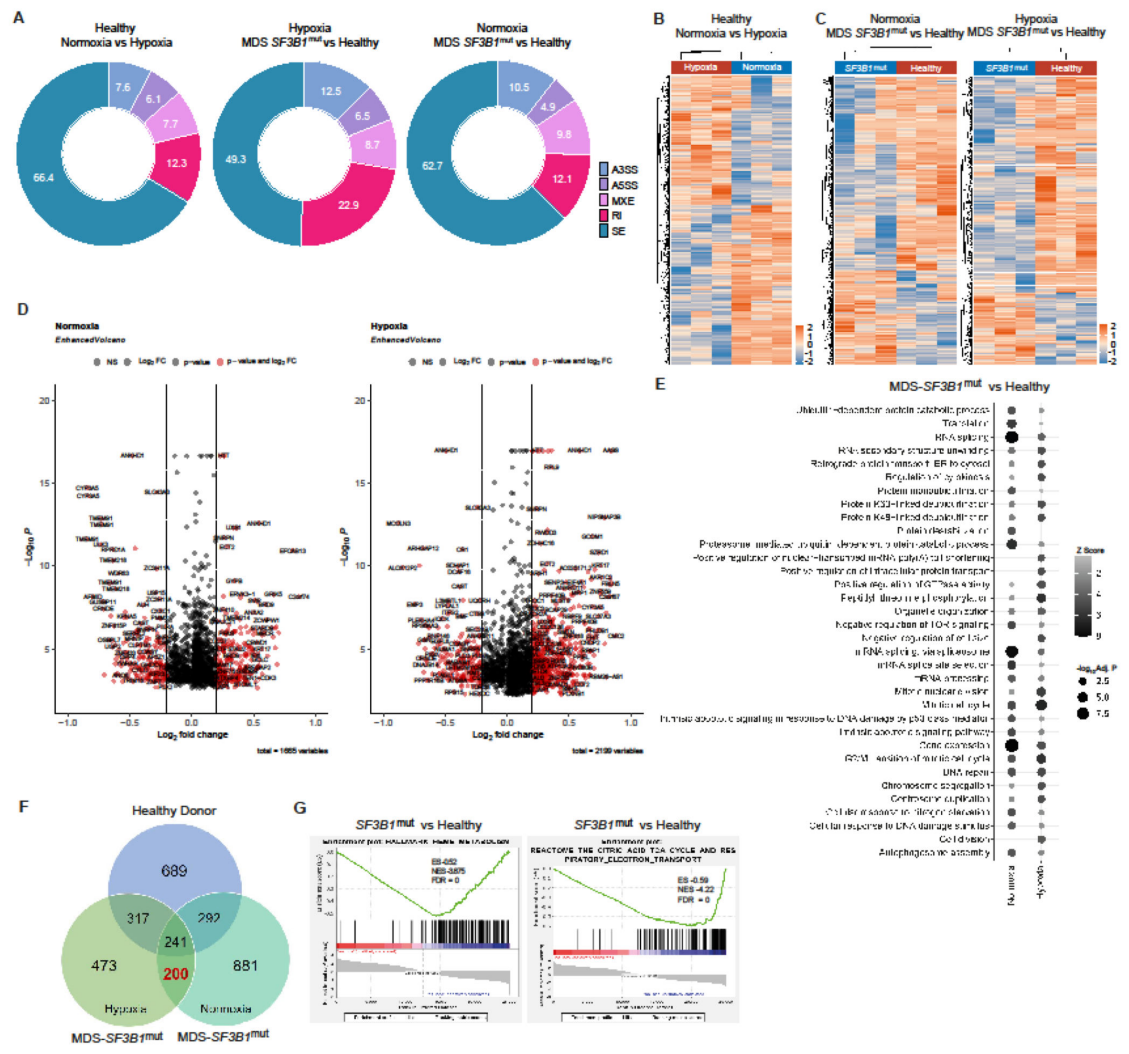
*SF3B1* mutations induce global mis-splicing of downstream genes in bone marrow haematopoietic stem and progenitor cells of patients with MDS-RS. **(A)** Pie chart representing the proportion of the mis-splicing events identified in the samples. **(B)** Heatmap of the differential splicing events captured between healthy donor colonies cultured under normoxic and hypoxic conditions. **(C)** Heatmap of the differential splicing events captured between healthy donor colonies and MDS *SF3B1*^mut^ colonies grown in normoxia (left panel) or hypoxia (right panel). **(D)** Volcano plot showing the genes that were differentially mis-spliced between MDS *SF3B1*^mut^ and HD samples in hypoxic and normoxic conditions with a *p*< 0.05 and Inclusion Level ≥ |0.2|. **(E)** Dotplot showing the top most significant Gene Ontology Biological Processes for each comparison (hypoxia MDS *SF3B1*^mut^ versus healthy donors, and normoxia MDS *SF3B1*^mut^ versus healthy donors) with adj. *p*< 0.05. **(F)** Venn diagram showing overlap between genes with significant splicing events identified in healthy donors and *SF3B1*^mut^ patients with MDS-RS. A total of 200 gene mis-splicing events that were present in patients with MDS *SF3B1*^mut^ but not in HDs were analyzed further. **(G)** GSEA analysis of the differentially expressed genes demonstrated defects in heme metabolism (top panel) and the citric acid cycle (TCA)/respiratory electron transport chain (bottom panel) in patients with MDS *SF3B1*^mut^.

**Figure 3:**
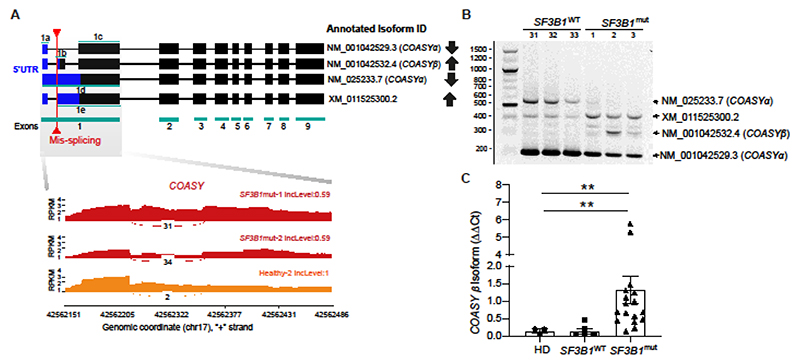
*SF3B1* mutations result in mis-splicing of *COASY* gene in patients with MDS-RS. **(A)** Exon structure of the different refseq annotated *COASY* isoforms. Area highlighted in blue depicts the mis-splicing target 5′ UTR region. The red arrows highlights the junction which is alternatively used in MDS *SF3B1*^mut^ cells. Sashimi plots underneath represent the read coverage in RPKM at exon-exon junction in COASY 5’UTR transcript for 2 patients with MDS *SF3B1*^mut^ (in red) and 1 healthy donor (in orange). Inclusion level (IncLevel) and numbers of reads covering the junction are displayed on the plot. **(B)** Representative gel image of *COASY* 5’UTR RT-PCR in MDS *SF3B1*^WT^ (n=3) and MDS *SF3B1*^mut^ (n=3) patients. **(C)** Quantitative PCR analysis of NM_001042532.4 isoform encoding COASY β in healthy donors (HD) (n=3), patients with MDS *SF3B1*^WT^ (n=5), and patients with MDS *SF3B1*^mut^ (n=17). Data presented here are the mean± S.E.M. ** *p<*0.01.

MDS haematopoietic cells evolve in both hypoxic and normoxic environments in the bone marrow and the blood stream, respectively. To capture the impact of *SF3B1* mutations in both environments, we performed our experiment under normoxic and hypoxic conditions. A cohort of 42 patients with MDS (15 SF3B1 wild type [WT]; 27 SF3B1 mutant) (Table S1) was used in this study. Bone marrow cells from three patients with MDS (*SF3B1^mut^* -H662Q/H662Q/K700E) and three age-matched healthy donors (HDs) were cultured in colony forming assay under normoxic (20% O_2_) and hypoxic (3% O_2_) conditions (30, 31) (Fig 1B-E). We then performed RNA-sequencing of the HSPC-derived colonies using 150 bp paired-end sequencing with an average read count of 138 million reads per sample (Fig. S1A). Differential splicing analysis using rMATS (32) identified 3,864 mis-spliced events common to the three MDS-RS samples, with a false discovery rate (FDR) <0.05. We observed an increase in A3SS events in *SF3B1^mut^* cells under normoxia and hypoxia (Fig 2A), consistent with previous studies (22). Unsupervised clustering of the mis-spliced events led to the clustering of *SF3B1*^mut^ samples distinctively from the cluster of HD samples with wildtype *SF3B1* (Fig 2B-C) (GEO accession number: GSE173108). The volcano plots of transcripts aberrantly spliced and ranked by degree of mis-splicing showed 1665 and 2199 events differentially regulated (significant events are represented in red, P <0.05; inclusion level >|0.2|), respectively, under normoxic and hypoxic conditions (Fig 2D). Gene Ontology pathway analysis of the mis-spliced genes that were enriched in MDS-RS samples compared to healthy donors upon hypoxia and normoxia revealed key cellular pathways affected such as translation and mRNA processing in patients with *SF3B1^mut^* (Fig 2E). Among the 2,845 mis-spliced genes, 200 were shared between hypoxic and normoxic conditions in *SF3B1^mut^* samples (Fig 2F & data files S1&S2). GSEA analysis of differential gene expression pointed to dampened heme metabolism as well as decreased activity of the tricarboxylic acid (TCA) cycle (Fig 2G). We therefore focused our analysis on splicing events that could affect these two pathways and this led to the identification of a mis-splicing in the coenzyme A (CoA) synthase (*COASY*) gene (Fig 3A-C, S1B-F). COASY catalyzes the last steps of the biosynthesis of CoA, a cofactor for 4% of all cellular enzymes (33). CoA is necessary for the production of acetyl-CoA for carbon entry into the TCA cycle and for the generation of succinyl-CoA, an essential substrate of aminolevulinic acid (ALA) synthase (ALAS2), the rate-limiting enzyme in heme biosynthesis (Fig. S2A). Germline mutations of *COASY* have been previously linked to an autosomal recessive disorder (neurodegeneration with brain iron accumulation, NBIA) (34–36) and pontocerebellar hypoplasia (37). NBIA ressembles some of the clinical manifestations of MDS erythropoiesis defects, including iron imbalance and heme biosynthesis defects. We therefore decided to investigate whether *COASY* mis-splicing could induce protein loss that would ultimately lead to a depletion of CoA and a decrease in succinyl-CoA abundance, thereby contributing to ineffective erythropoiesis in patients with MDS *SF3B1*^mut^.

### *COASY* transcript mis-splicing induces protein loss and CoA synthesis deficiency in *SF3B1*^mut^ cells

*COASY* mis-splicing occurs within the 5’UTR and is categorized as an alternative 3’ splice site (A3SS) with an inclusion level of 38.7%. This mis-splicing event was associated with a switch of alternative transcripts that sees the transcript NM_001042532.4 (COASY beta) and XM_011525300.2 become the dominant isoforms in patients with MDS *SF3B1*^mut^ over the canonical NM_001042523.9 isoform encoding COASYα (Fig 3B). RT-qPCR performed on a discovery cohort of 25 patient samples (3 HDs, 5 MDS *SF3B1* WT, and 17 MDS *SF3B1^mut^)* demonstrated that the beta isoform was exclusively seen in patients with mutated *SF3B1* (Fig 3C). These results were confirmed in an independent validation cohort (10 MDS *SF3B1* WT and 8 MDS *SF3B1^mut^)* (Fig. S1E). Significant changes were also observed by RT-qPCR for the XM *COASY* isoform (p<0.01) whereas the alpha isoform of *COASY* remained unchanged (Fig 3B & Fig. S1F).

**Figure 4:**
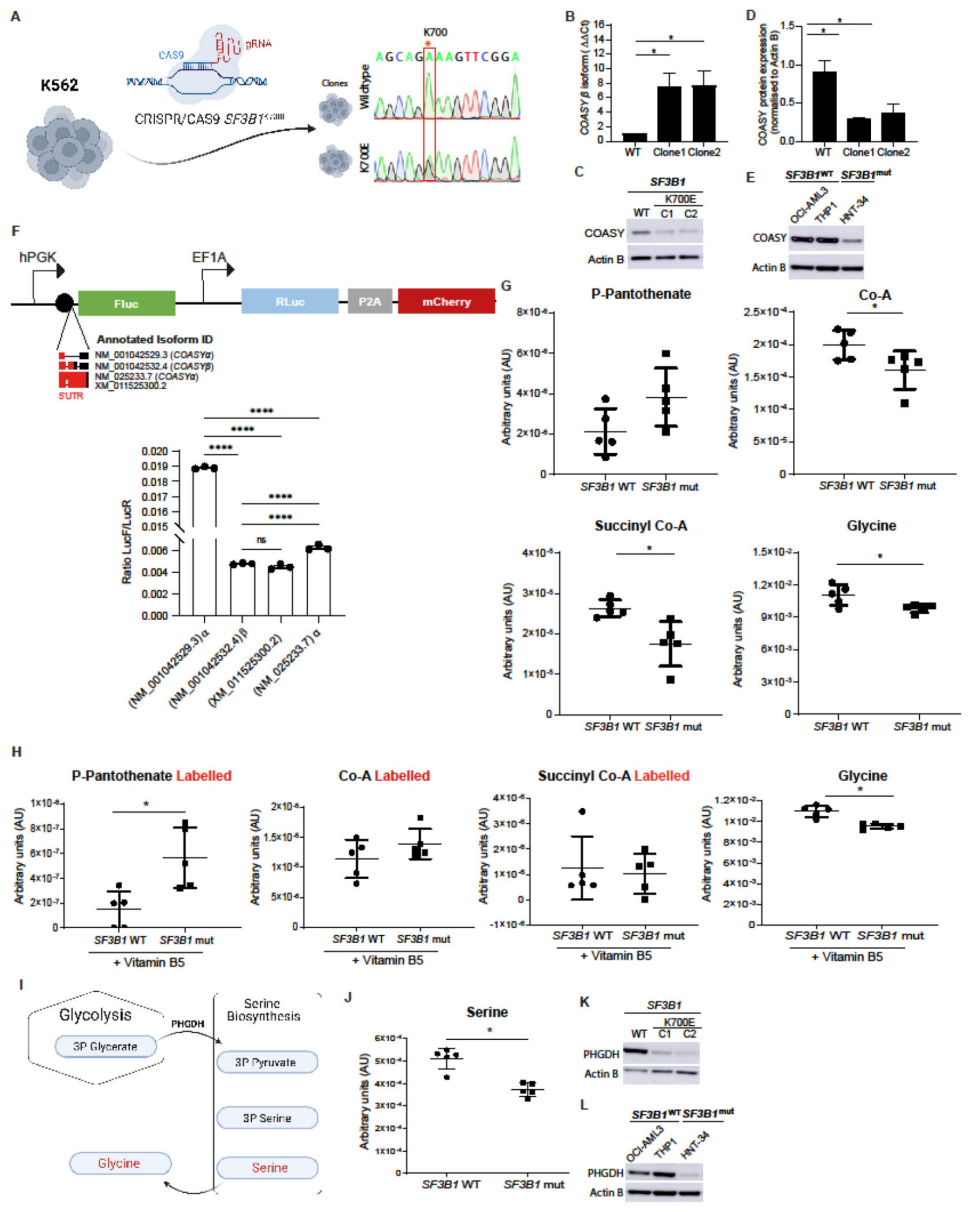
*COASY* transcript mis-splicing induces protein loss and reduction of CoA synthesis in *SF3B1*^mut^ cells. **(A)** CRISPR-cas9 editing strategy used to insert *SF3B1* heterozygous K700E mutation in K562 cell line. **(B)** Quantitative PCR analysis of NM_001042532.4 isoforms, encoding COASY β protein, in K562 *SF3B1*^wt^ and K562 *SF3B1*^mut^ clones (Clone1 and Clone2) (n=3). **(C)** Representative Western blot showing COASY protein abundance in K562 *SF3B1*^wt^ and K562 *SF3B1*^mut^ clones (C1 and C2). Actin B protein was used for all Western blots as a loading control. **(D)** Quantification of COASY protein expression in K562 *SF3B1*^wt^ and K562 *SF3B1*^mut^ clones (Clone1 and Clone2). Data here are normalized to Actin B (n=3). **(E)** Representative Western blot showing COASY protein expression in *SF3B1^wt^* AML cell lines (OCI AML3 and THP-1) and *SF3B1*^mut^ AML cell lines (HNT-34). **(F)** Schematic representing the dual luciferase vector pDualLuc (top panel). Firefly and Renilla luciferases (FLuc and RLuc) are under the control of two independent promoters, hPGK and EF1A, respectively. The 5’UTR from the 4 different *COASY* isoforms (NM_001042529.3, NM_001042532.4, NM_025233.7, XM_011525300.2) were cloned upstream of *FLuC*. Rluc expression was used for normalisation. We quantified the impact on translation of the 5’UTR of each isoform by dual luciferase assay (n=3) (bottom panel). **(G)** LC-MS/MS analysis of phospho-pantothenate, CoA, succinyl-CoA, and glycine concentrations in K562 *SF3B1*^wt^ and K562 *SF3B1*^mut^ (N=5) **(H)** LC-MS/MS analysis of phosphopantothenate, CoA, succinyl-CoA, and glycine abundance in K562 *SF3B1*^wt^ and K562 *SF3B1*^mut^. Metabolites were labelled by incubating cells with 0.25mg/L vitamin B5 stable isotope (13C6, 15N2) for 24h (N=5). **(I)** Schematic representation of the serine-glycine synthesis pathway. 3-phosphoglycerate from glycolysis is converted into 3-phospho-pyruvate by the phosphoglycerate dehydrogenase enzyme (PHGDH). Successively 3-phospho-pyruvate is converted into 3-phosphoserine, serine, and glycine. **(J)** LC-MS/MS analysis of serine abundance in K562 *SF3B1*^wt^ and K562 *SF3B1*^mut^ (N=5). **(K)** Representative Western blot showing PHGDH protein abundance in K562 *SF3B1*^wt^ and K562 *SF3B1*^mut^ (Clone1 and Clone2) (n=3). **(L)** Representative Western blot showing PHGDH protein abundance in *SF3B1*^wt^ AML cell lines (OCI AML3 and THP-1) and *SF3B1*^mut^ AML cell lines (HNT-34). Data presented are the mean± S.D. **** *p<0.001*, * *p<0.05*.

Using the CRISPR/Cas9 system, we introduced the most common *SF3B1* heterozygous mutation (K700E) into the K562 cell line (Fig 4A). This resulted in 5’UTR mis-splicing of *COASY* that exactly replicated the profile observed in patients with MDS-RS and recapitulated other previously reported mis-splicing events (Fig 4B & Fig. S2B-C). This isoform switch resulted in ≈60% loss of COASY protein expression (Fig 4C-D). This finding was confirmed in HNT-34 (a CMML/AML patient derived cell line) that harbors the *SF3B1*^K700E^ mutation (Fig 4E & Fig. S2D). Identical results were observed in K562 cells expressing *SF3B1*^K700EFLAG^ under the control of a doxycycline inducible promoter(38) (Fig. S). Notably, Western blot analysis of the primary CD34^+^ cells isolated from one healthy donor, four *SF3B1* WT and three *SF3B1* mutant MDS-RS samples from patients confirmed the loss of COASY expression in *SF3B1* mutated samples (Fig. S2F).

To better understand the mechanism underlying the loss of COASY protein, we measured *COASY* mRNA abundance in *SF3B1*^mut^ cells and assessed the impact of each 5’UTR on protein synthesis of different *COASY* isoforms. RT-qPCR in *SF3B1*^mut^ cells showed no difference of *COASY* total mRNA abundance (Fig. S2G), suggesting that the regulation does not occur at the transcriptional level. However, cloning of different 5’UTR of *COASY* isoforms (detected in patient MDS-RS cells) in a dual luciferase vector demonstrated that only the isoform NM_001042529.3 (COASY α) allowed an efficient translation of the protein, whereas all three other isoforms impeded *COASY* translation (Fig 4F).

Next, to assess whether *COASY* mis-splicing leads to measurable defects in the TCA cycle and metabolites essential for heme biosynthesis (Fig S2A), we performed LC-MS/MS metabolic analysis. This demonstrated an impairment of the TCA cycle with a significant decrease (p<0.05) in CoA, succinyl-CoA, and, unexpectedly, in glycine, a co-substrate of ALAS2 with succinyl-CoA (Fig 4G & Fig. S3A). Although CoA and succinyl-CoA concentrations were rescued by saturating the remaining activity of COASY with its upstream substrate vitamin B5, this did not rescue the glycine depletion (Fig 4H). Glycine is produced from serine by two enzymes, SHMT1 (serine hydroxymethyltransferase 1, cytoplasmic) and SHMT2 (serine hydroxymethyltransferase 2, mitochondrial). PHGDH (phosphoglycerate dehydrogenase) converts the glycolytic intermediate 3-phospho-glycerate into 3-phosphohydroxy-pyruvate, which is then converted into 3-phosphoserine and serine by PSAT1 (phosphoserine aminotransferase 1) and PSPH (phosphoserine phosphatase), respectively (Fig 4I). In line with this observation, we noted a marked decrease in serine abundance that coincided with mis-splicing and a loss of the protein PHGDH (Fig 4J-L & Fig. S3B). This disruption in serine is likely to be responsible for the reduction in glycine. These data are in agreement with recent studies that reported *PHGDH* mis-splicing in patients with *SF3B1^mut^* MDS and in a breast cancer model (39, 40).

### COASY deficiency impairs erythroid differentiation in human primary cells

**Figure 5:**
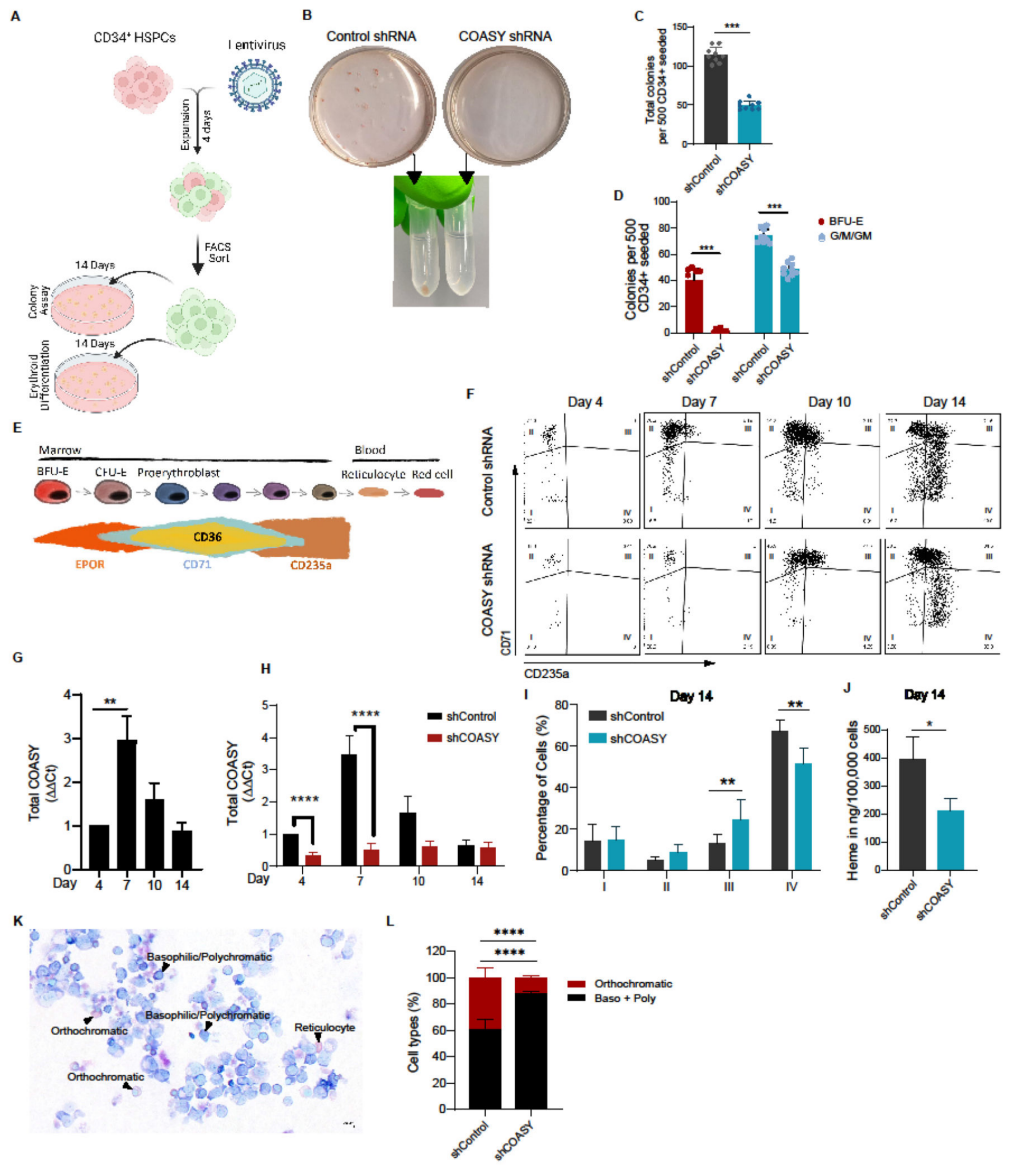
*COASY* deficiency impairs erythroid differentiation. **(A)** *COASY* knock-down strategy used in healthy donor CD34^+^ umbilical cord blood cells. Cells were transduced with shRNA-tagged eGFP reporter against *COASY* (shCOASY) or a scrambled sequence (shControl) and expanded for 4 days before FACS sorting of CD34^+^/GFP^+^ cells. shCOASY and shControl CD34^+^ cells were seeded in methylcellulose to assess colony-forming potential or in liquid culture for erythroid differentiation. **(B)** Pictures of colonies obtained with shControl cells (left) and shCOASY transduced CD34^+^ cells (right), at day 14. Progenitor cells from colony-forming assay were harvested and pellets are shown as representative picture. **(C)** Total colonies per 500 CD34^+^ cells seeded in methylcellulose for shControl and shCOASY KD CD34^+^ cells (n=3), at day 14. **(D)** BFU-E and G/M/GM colonies per 500 CD34^+^ cells seeded in methylcellulose for control and shCOASY KD HSPCs cells (n=3), at day 14. **(E)** Representation of the different erythroid differentiation stages and associated surface markers acquired during the differentiation process. **(F)** Flow cytometry analysis of CD71/CD235a acquisition in shControl and shCOASY KD cells at day 4, 7, 10, and 14 of erythroid differentiation. **(G)** Quantitative PCR analysis of total COASY expression in CD34^+^ HSPCs cells at day 4, 7, 10, and 14 of erythroid differentiation (n=3). **(H)** Quantitative PCR analysis of total *COASY* expression in CD34^+^ HSPCs cells transduced with shControl or shCOASY1 at day 4, 7, 10, and 14 of erythroid differentiation (n=3). **(I)** Percentage of cells in quadrants I, II, III and IV, as depicted in (F), were quantified for shControl and shCOASY KD HSPCs cells at day 14 of erythroid differentiation (n=3). **(J)** Heme quantification for shControl and shCOASY KD HSPCs cells at day 14 of erythroid differentiation (n=3). **(K)** Representative image for quantification of erythroid cell morphology using Giemsa staining of CD34^+^ HSPCs cells after erythroid differentiation, at day 10. Scale bar, 20μm. **(L)** Quantification of types of erythroid cells based on morphological features using Giemsa staining of shControl or shCOASY CD34^+^ HSPCs cells following erythroid differentiation, at day 10. Basophilic/polychromatic and orthochromatic erythroblasts were counted from 10 representative fields for each replicate (n=3). Data are the mean ±S.E.M. ****p<0.001, ***p<0.005, **p<0.01, *p<0.05.

To investigate whether depletion in succinyl-CoA induced by the loss of COASY could affect erythropoiesis, we silenced *COASY* in CD34^+^ HSPCs using a lentiviral approach (Fig 5A & Fig. S3C-D). Direct silencing of *COASY* resulted in significant depletion of CoA (p<0.0001) (Fig. S3E) and this was consistent with data obtained from our *SF3B1* mutant model (Fig. S3F). After *COASY* knockdown, clonogenic capacity of the CD34^+^ HSPCs was significantly reduced (p<0.005), with a substantial impact on Burst Forming Unit-Erythroid (BFU-E) activity (Fig 5B-D & Fig. S4A-D). These results prompted us to analyze *COASY* gene expression in the most immature haematopoietic compartments (Fig. S5A-B) and during erythroid differentiation (Fig 5E-L). Notably, *COASY* showed a peak of expression at day 7 (Fig 5G) that precedes the peak of heme production observed at day 10 by another study (41).

Furthermore, silencing of *COASY* in CD34^+^ HSPCs caused significant delays in erythroid differentiation (p<0.01), with accumulation of erythroblasts CD71^+^CD235a^+^, fewer mature cells than CD71^-^CD235a^+^ (Fig 5I), and a decrease in 5-aminolevulinate and heme production (Fig. S5C & Fig 5J) that resembles the clinical phenotype of primary MDS-RS samples from patients. (41) Giemsa staining on cells with *COASY* knockdown after erythroid differentiation showed an accumulation of less differentiated (basophilic/polychromatic) erythroblastsand fewer differentiated (orthochromatic) erythroblasts compared to control cells (Fig 5K-L). Our data reveals the role of COASY in erythroid differentiation of human HSPCs.

### Vitamin B5 and succinyl-CoA rescue erythroid differentiation defects observed in MDS-RS *SF3B1*^mut^

**Figure 6:**
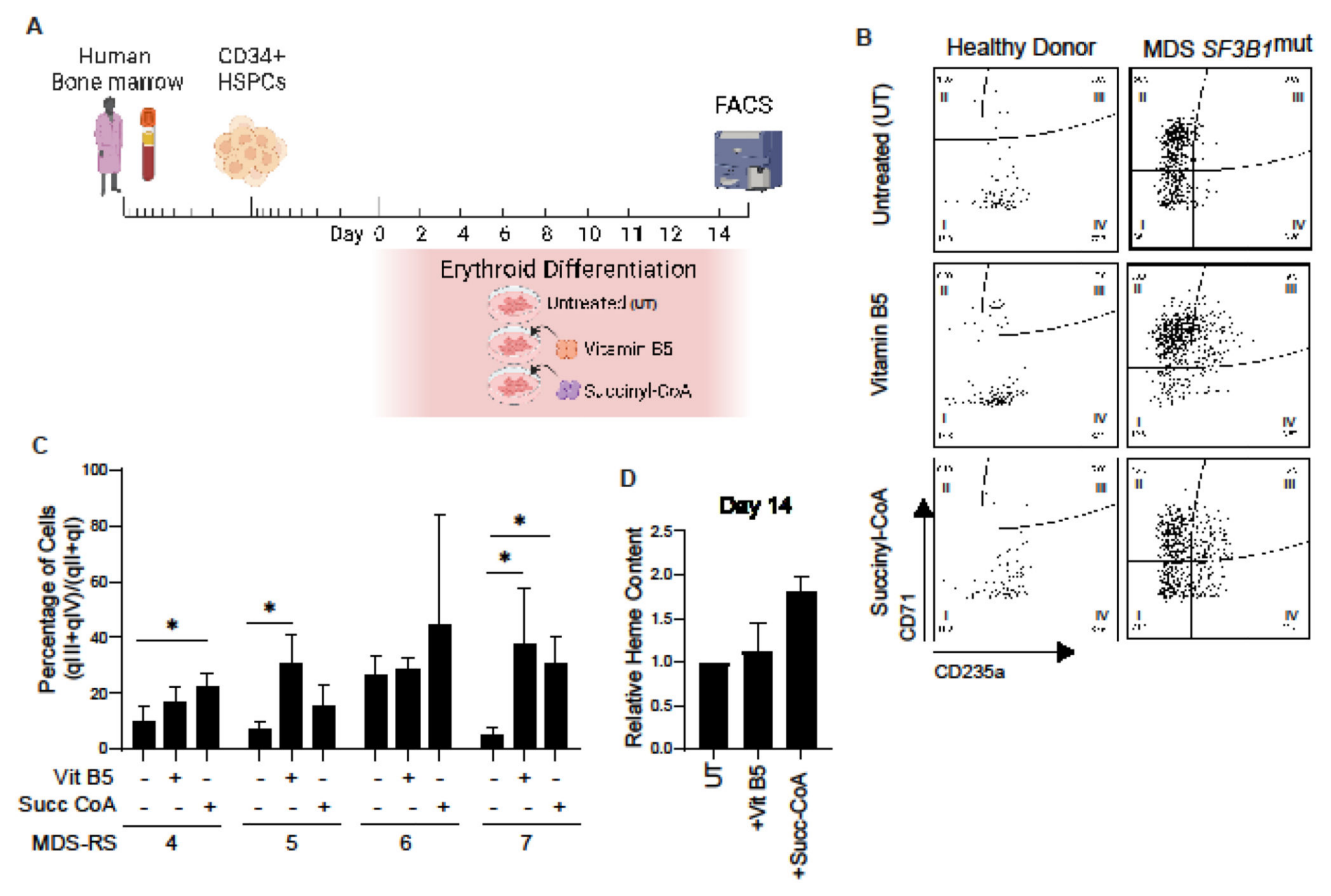
Vitamin B5 and succinyl-CoA rescue the erythroid differentiation defect observed in MDS-RS *SF3B1*^mut^ cells. **(A)** Schematics of the strategy used to rescue erythroid differentiation defect in MDS *SF3B1*^mut^ cells. CD34^+^ cells were isolated from MDS *SF3B1*^mut^ bone marrow MNCs and cultured in erythroid differentiation medium, supplemented with/without additional vitamin B5 or succinyl-CoA, for 14 days and then analyzed by flow cytometry. **(B)** Flow cytometry analysis of CD71/CD235a acquisition in BM CD34^+^ from adult healthy donor and MDS *SF3B1*^mut^ cells from patients, treated with/without additional vitamin B5 or succinyl-CoA, at day 14 of erythroid differentiation. **(C)** Percentage of cells during erythroid differentiation. Data are represented as the ratio [(quadrant III + quadrant IV) / (quadrants II + quadrants I)] of cells, as quadrants are depicted in (B), and quantified for MDS *SF3B1*^mut^ cells from patients, treated with/without vitamin B5 or succinyl-CoA, at day 14 of erythroid differentiation. 4 independent patients’ samples (MDS-RS #4, #5, #6, #7) were analyzed with a minimum of 3 replicates for each condition. Data presented here is the mean± S.E.M. *p<0.05. **(D)** Relative heme content, expressed as fold change, per 200,000 MDS *SF3B1*^mut^ cells treated with vitamin B5 or succinyl-CoA, compared to untreated at day 14 of erythroid differentiation (n=2).

Given the critical role revealed for COASY during erythropoiesis, we assessed whether the COASY/CoA/succinyl-CoA axis could be targeted in primary patients with MDS-RS to rescue ineffective erythropoiesis. With the rationale that patients *SF3B1*^mut^ cells would still express 40% of COASY enzyme, in our erythroid differentiation model we treated primary CD34^+^ patient cells (isolated from a patient with MDS-RS or healthy donor) with upstream substrates COASY, vitamin B5, or its downstream by-product succinyl-CoA (Fig 6A). Consistent with a previous report, (41) MDS-RS cells treated with erythropoietin were blocked in differentiation after 14 days and amassed in CD71^-^CD235a^-^ and CD71^+^CD235a^-^ fractions, and only a few cells matured into CD71^-^CD235a^+^ cells (Fig 6B). Treatment of MDS-RS cells with vitamin B5 significantly increased maturation of CD71^+^CD235a^+^ and CD71^-^CD235a^+^ (p<0.05), whereas treatment with succinyl-CoA rescued erythroblasts maturation and heme production in patients with MDS-RS (Fig 6C-D & Fig. S6A-E). It is important to note that as a result of the treatment with either of the substrates, no changes of the different erythroid population were observed in the healthy donor BM HSPCs. To demonstrate that it is succinyl-CoA and not the succinate component that rescues erythroid lineage differentiation, umbilical cord blood (UCB) HSPCs cells silenced for *COASY* were treated with succinyl-CoA, succinate, or vehicle. Notably, only succinyl-CoA rescued the phenotypic and morphologic differentiation of erythroid cells silenced for *COASY* (Fig. S6F-G). Altogether, our results demonstrate that vitamin B5 and succinyl-CoA can override the erythroid blockage in patients with MDS-RS and increase erythroid cells maturation by stimulating heme production in erythroid progenitor cells (Fig. S7).

## Discussion

Anemia is the most common hematologic manifestation of MDS. Acquisition of mutation in the splicing factor SF3B1 is a key event in the establishment and progression of MDS and other cancers. Some progress has been made in dissecting the downstream effects of *SF3B1* mutations on dysregulated RNA splicing (17, 42–44). However, determining the mechanism(s) driving ineffective erythropoiesis in *SF3B1* mutant HSPC clones remains a major challenge and contributes to the slow progress towards developing an effective therapy.

Here, we have identified a mis-splicing in the transcript encoding the coenzyme A synthase, a core bifunctional enzyme that catalyzes the fourth and fifth sequential steps of CoA biosynthetic pathway, in *SF3B1* mutant patients with MDS-RS. Germline mutations of *COASY* have been reported in an autosomal recessive neurodegenerative disorder called neurodegeneration with brain iron accumulation (NBIA). Modelling of the human *COASY* mutation in yeast revealed defects in mitochondrial function and iron metabolism that resemble MDS phenotype (45). Using a variety of omics and functional assays, including splicing analysis, translation reporters, metabolomics, silencing in primary human cells, colonies, and differentiation assays we revealed a critical role of COASY in regulating normal BM erythropoiesis through control of succinyl-coA availability during erythroid differentiation. Silencing of *COASY* in human primary HSPCs impaired erythroid clonogenic capacities and delays erythroid differentiation. The peak of *COASY* transcription during erythroid maturation preceded the accumulation of heme, suggesting that the increase in *COASY* expression is necessary to supply succinyl-CoA in sufficient quantity for heme synthesis. Although all cells synthesize heme, it is worth noting that we observed a mild effect on granulocytic and monocytic colony formation upon *COASY* knockdown. Although in healthy HSPCs *COASY* silencing alone is capable of disrupting erythroid differentiation, its role in other hematopoietic lineages, in stem and progenitor cells, and its potential impact to wider cytopenia need to be further investigated.

Alternative splicing of coding and non-coding regions (including untranslated regions) is a fundamental regulatory mechanism at the crossroads between transcription and translation that governs mRNA stability, localization, or translation (46, 47); it may be noted in passing that >95% of human genes’ pre-mRNA are spliced (48–51). The importance of such a mechanism is well illustrated by the fact that at least 15% of mutations that cause genetic disease affect pre-mRNA splicing (52). In line with these studies, we have demonstrated that mis-splicing of the 5’UTR of *COASY* by *SF3B1^mut^* impedes its translation. The analysis of 42 MDS primary patient samples, including 25 samples harboring mutations in *SF3B1*, showed a systematic increase of the alternative splice isoform of COASY NM_001042532.4 that encodes COASYβ, although *COASY* total gene expression remained unchanged. Analysis of our patient RNA-seq data revealed a profound disruption of heme metabolism and TCA cycle pathways. *In vitro* modelling of *SF3B1* mutated cells and metabolomic analysis confirmed that *COASY*’ switch of alternative isoform causes a depletion of CoA and succinyl-CoA. Furthermore, *COASY* knockdown in healthy donor HSPCs dampened heme synthesis during erythroid differentiation. Our results demonstrate that the partial loss of the coenzyme A synthase in patients with MDS-RS leads to a substantialdisruption in the production of heme synthesis in progenitors undergoing erythroid differentiation, and thus contributes to the accumulation of undifferentiated erythroblasts in these patients.

Erythroid dysregulation is a common feature of inherited sideroblastic anemias, where inherited mutations of several genes involved in heme biosynthesis, iron-sulfur (Fe-S) cluster biogenesis, Fe-S cluster transport, and mitochondrial metabolism have been reported (53). In MDS-RS, maturation arrest occurs in an early erythroid precursor (54) which resembles the phenotype of loss of colonies and erythroid differentiation delays that we observed upon silencing of *COASY* in HSPCs.

Next, we tested whether the treatment of *SF3B1* mutant cells with either vitamin B5 or succinyl-CoA could rescue their erythroid differentiation *in vitro*. *De facto*, supplementing media with vitamin B5 saturated the remaining COASY activity and reinstated CoA as well as succinyl-CoA concentratons, whereas using succinyl-CoA greatly improved erythroid differentiation by directly increasing ALAS2’ substrate availability. It is important to note that healthy donor HSPCs that underwent the same treatment regimen followed a normal erythropoietic differentiation course. Vitamin B5 deficiency is rare in humans, and most of the data regarding the consequences of its deficiency have been generated from animal experiments. For instance, in macaques vitamin B5 deficiency causes anaemia, irritability, and fatigue due to decreased synthesis of heme (55). Therefore, considering that vitamin B5 deficiency leads to erythroid dysregulation, it is reasonable to envisage that sustaining vitamin B5 intracellular concentrations could be used to saturate the remaining COASY activity and prevent its deleterious effects on CoA metabolism. Our data provides a rationale for screening for vitamin B5, CoA, and succinyl-CoA concentrations in MDS-RS cases, and potentially in other conditions where anemia is a common feature.

Altogether, our findings may open therapeutic avenues for preventing iron accumulation in patients with MDS-RS. Notably, the detection of the alternative splice form of *COASY* by RT-qPCR could form the basis of a diagnostic test in suspected cases of MDS-RS. Furthermore, the COASY/CoA/succinyl-CoA axis represents a potential therapeutic target that is highly relevant for the treatment of ineffective erythropoiesis in patients with MDS-RS. Although succinyl-CoA would require further pharmacokinetic/absorption analyses to determine safety, feasibility studies have already shown that pantothenic acid/vitamin B5 is safe and well tolerated when administered orally in patients (56). Therefore, vitamin B5 represents an attractive agent to combine with existing treatments in order to increase erythroid maturation and delay red blood cell transfusion dependency in patients with MDS-RS.

## Material and Methods

### Primary samples

Mononuclear cells from MDS Patients (n=42) were received from Barts NHS Trust, University of Manchester, University Hospital of Leipzig, and University of Brest, and were analysed under the research ethics protocol (05/Q0605/140). Consent form were obtained for all samples according to local tissue bank guidelines. Patients’ clinical characteristics are detailed in Table S1. The clinical variables for all patients were ascertained at the time of sample collection. Targeted DNA mutations data were available for all patients. Adult bone marrow healthy donor cells and cord blood samples were purchased from Stem cell technologies and Anthony Nolan, respectively.

### Cell line culture conditions

K562, OCI-AML3, THP-1, and HEK-293 cells were purchased from ATCC. HNT-34 cell line was purchased from DSMZ. Human STR profiling was confirmed in all cell lines. K562, OCI-AML3, THP-1, and HNT-34 cell lines were cultured according to supplier’s instructions in RPMI (ThermoFisher) supplemented with 10% FBS (Sigma) and 1% Penicillin/Streptomycin (ThermoFisher). HEK-293 cells were cultured in DMEM (ThermoFisher) supplemented with 10% FBS and 1% P/S.

### K562 *SF3B1* mutant cell line

A *SF3B1* K700E mutation was introduced in K562 cells by CRISPR/Cas9 editing. sgRNA [AGTTCGGACCATCAGTGCTT (TGG)] was designed with CRISPOR online tool and purchased from Synthego. sgRNA was then cloned in px458 vector (Addgene). 200,000 K562 cells were co-transfected with 2μg of the plasmid and 100pmol of ssODN (GGTAATGTTGGGGCATAGTTAAAACCTGTGTTTAGTTTTGTAGGTCTTGTGGATGAGCAG CAGGAAGTTCGGACCATCAGTGCTTTAGCCATTGCTGCCTTGGCTGAAGCAGCAACTCCT, Sigma) using the Neon system (1450V, 10ms, 3 pulses). 24 hours after the transfection, cells were stained with DAPI (4,6, diamidino-2-phenylindole) and GFP+ live cells were sorted using FACS ARIA (BD Biosciences). Cells were cultured using RPMI (ThermoFisher) supplemented with 10% FBS (Sigma) and 1% Penicillin/Streptomycin (ThermoFisher) for 7 days. Following on, single cells were FACS sorted into a 96-well plate and individual clones were expanded. After expansion, individual clones were genotyped using a T7 Assay (NEB) with the following primers: Forward (5’-AGGTACACACACAGCCTGTCC-3’) and Reverse (5’-TGGTGGATTTACCTTTCCTCTG-3’). *SF3B1* K700E heterozygous mutation in the positive clones was confirmed by Sanger sequencing. Doxycycline inducible K562 *SF3B1*^K700E^ cells were a kind gift from Dr Delphine Bernard (38).

### MNCs and CD34^+^ isolation from human bone marrow cells

Mononuclear cells (MNCs) were isolated from the bone marrow or cord blood cells by centrifugation using Ficoll-PaqueTM PLUS (GE Healthcare Life Sciences). CD34^+^ cell enrichment was performed using EasySep Human CD34 Positive Selection Kit II (StemCell Technologies) according to the manufacturer’s instructions.

### Colony forming assay

1,500 CD34^+^ HSPCs were seeded in 0.5 mL methocult H4434 (StemCell Technologies) supplemented with 1% penicillin/streptomycin (Sigma-Aldrich) in a 24-well plate. Colonies were grown under normoxic (37°C and 20% O_2_) or hypoxic conditions (37°C and 3% O_2_). After 14 days of culture, colonies were scored and then harvested. Cells were washed with PBS and subsequently used for transcriptomic analysis.

### RNA sequencing library preparation

Cells harvested from colony forming assay were washed in PBS and lysed in TRI Reagent (Zymo Research). RNA extraction was performed using Direct-zol RNA Kits (Zymo Research) according to the protocol of the manufacturer. RNA concentration and quality were determined with the Agilent 2100 Bioanalyzer system using the Eukaryote Total RNA Pico Assay kit. RNA sequencing libraries were prepared using Takara SMARTer Stranded Total RNA-Seq Kit v2 - Pico Input Mammalian kit according to the manufacturer’s instructions. The quality and concentration of final libraries were assessed using Agilent 2100 Bioanalyzer system and Qubit DNA HS assay, respectively.

### RNA sequencing analysis

Sequencing of the RNA libraries was performed on Novagene Illumina NovaSeq6000 platform. Sequencing generated an average ~138 million paired-end reads of 150 bp in length for each sample. Fastqc was performed using fastqc version 0.11.5 and adapter sequences were trimmed using cutadapt version 2.1. Raw reads were mapped to the human genome (hg19, Genome Reference Consortium GRCh37) using HISAT2 version 2.1.0 (PMID:25751142). The number of reads aligned to the exonic region of each gene were counted using HTSeq version 0.11.2 (PMID:25260700) based on the Ensembl annotation. Only genes that achieved at least one read count per million reads (cpm) in at least five samples were included for analysis. Conditional quantile normalisation (cqn) (PMID: 22285995) was performed counting for gene length and GC content, and a log2 transformed RPKM expression matrix was generated. Differential expression analysis was performed using the ‘limma’ R package and voom normalisation (PMID:25605792). Gene-set enrichment analysis (GSEA) of these genes was performed using GSEA software (PMID :16199517) on the publicly available bioinformatics platform GenePattern (PMID:16642009) for Gene Ontology Biological Processes (c5.bp.v6.2.symbols.gmt) and Canonical Pathways (c2.cp.v6.2.symbols.gmt). Heatmaps illustrating the expression pattern of the genes were generated using R package ComplexHeatmap. Row clustering was performed on Euclidean distance using the “complete” clustering method. Volcano plots were generated using R package EnhancedVolcano. Differential splicing was examined using rMATS version 4.0.2. Aberrant splicing events associated with the mutated phenotype were identified on the basis of significant events identified by rMATS comparing healthy donor samples with MDS samples (including hypoxia and normoxia conditions, FDR < 0.05). Enrichment analysis of genes with significant splicing events was performed using the function dEnricher of the dnet R package. GSEA pre-ranked analysis of the differential gene expression data (HD vs MDS samples) was performed according to MSigDB (version 7.1) with default settings. Dotplot heatmaps were generated by selecting the top 20 most significant gene ontology biological processes for each comparison (hypoxia MDS^*SF3B1mut*^ versus healthy and normoxia MDS^*SF3B1mut*^ versus healthy) with adjp < 0.05. RNA-Seq data have been deposited in Gene Expression Omnibus (GEO) under the accession number GSE173108.

### Lentivirus production in HEK 293

Two lentiviral vectors, shRNA_COASY#1 (AGGCCTTTGGAACAGATATTC) and shRNA_COASY#2 (CCTACCCAACACGCTGGTATT) targeting the human COASY gene, and one control against Lac (shRNA_Control) (CCTAAGGTTAAGTCGCCCTCG) were purchased from Vectorbuilder. All vectors expressed GFP as a reporter gene. Viral particles for all the shRNAs were produced by transient CaCl2 transfection of HEK293 cells and harvested by ultracentrifigution.

### CD34^+^ UCB cells transduction

UCB CD34^+^ HSPCs were stimulated using StemSpan medium (Stem cell Technologies) supplemented with cytokines (150 ng/ml SCF,, 150 ng/ml Flt-3, 10 ng/ml IL-6, 25 ng/ml G-CSF, and 20 ng/ml TPO, all from PeproTech) and 1% HEPES (Sigma-Aldrich) for 4-6 hours. Virus particles were then added to the stimulated cells (multiplicity of infection, MOI=30) and cells were incubated (37°C) overnight. Cells were washed and resuspended in expansion medium supplemented with cytokines (150 ng/ml SCF, 150 ng/ml Flt-3, 10 ng/ml IL-6, 25 ng/ml G-CSF, and 20 ng/ml TPO) and 1% HEPES. Cells were expanded for 4 days. Following on, cells were stained with an antibody specific for human CD34 antigen. DAPI (4,6, diamidino-2-phenylindole, Sigma-Aldrich) staining was used to exclude dead cells and debris from the analysis. CD34^+^GFP^+^ cells were FACS sorted and then used downstream assays.

### Erythroid differentiation and rescue experiments

CD34^+^ HSPCs from patients with *SF3B1*^mut^ MDS, healthy donors’ bone marrow or transduced UCB (CD34^+^GFP^+^) was cultured in erythroid differentiation medium (SCF 25ng/mL, EPO 3U/mL, and IGF1 50ng/mL, all from PeproTech) for 14 days. Cells were supplemented or not with 250nM of Succinyl-CoA (Sigma), 0.25mg/L vitamin B5 (Generon), or 250nM succinate (Merck) every other day for 14 days. For immunophenotyping, cells were stained with antibodies specific for human antigens (CD71 PE RRID:AB_2201481; CD235a APC/Cyanine7, RRID:AB_ 2650977) and DAPI. Cells were then analysed using a Fortessa flow cytometer (BD Biosciences) at day 4, 7, 10 and 14 for the erythroid differentiation with CD34^+^ UCB cells and at day 14 for experiments with MDS-RS patients’ samples.

### Giemsa Staining

10,000-100,000 cells per condition were collected for staining procedure and washed with PBS. 150uL of cells in PBS were spun down on slides with the Shandon Cytospin 3 at 800RPM for 3 min, and slides were rapidly fixed in ice cold methanol for 15 min at 4C. The slides were then consecutively flooded in May-Grunwald solution (Generon) for 5 min at room temperature, in PBS, in Giemsa solution (abcam) for 20 min at room temperature, and in water. The slides were dried before mounting in resin with the DPX mounting medium (VWR). Pictures were acquired with the Pannoramic 250 High Throughput Scanner (3D HISTECH).

### Phenotyping and isolation of CD34^+^ cells, Hematopoietic Stem cells (HSCs), Multipotent Progenitors (MPPs), Multi-Lymphoid Progenitors (MLPs), Common Myeloid Progenitors (CMPs), Granulocyte-Monocyte Progenitors (GMPs), and Megakaryocyte–Erythroid Progenitors (MEPs)

Frozen UCB cells were thawed and selected using an Easysep Human CD34 positive selection kit and Easysep magnet (StemCell Technologies) according to the manufacturer's instructions. CD34+ cells were stained with the following antibodies: CD34 PerCP-Cy5.5 (BD Biosciences, 347203) and CD135 BV711 (BD Biosciences, 563908), CD38 PE-Cy7 (eBioscience, 25-0388-42), CD45RA APC-Alexa eFluor 780 (eBioscience, 47-0458-42), CD90 APC (eBioscience, 17-0909-42). Cells were sorted as followed: HSC CD34^+^CD38^-^CD45RA^-^CD90^+^, MPP CD34^+^CD38^-^CD45RA^-^CD90^-^MLP CD34^+^CD38^-^CD45RA^+^, CMP CD34^+^CD38^+^CD45RA^-^CD135^+^, GMP CD34^+^CD38^+^CD45RA^+^CD135^+^ and MEP CD34^+^CD38^+^CD45RA^-^CD135^-^. DAPI was added to the cell suspension before sorting to exclude dead cells. Cells were sorted on a BD InfluxTM cell sorter operating in 6-way purity sort mode and collected into 1.5 ml microfuge tubes.

### Sample preparation for LC-MS/MS

1.5 million K562 cells were incubated with stable isotope 0.25mg/L calcium pantothenate (13C6, 15N2) (Merck), 0.3g/L glutamine (13C5) (Merck) or vehicle (H_2_0) for 4 hours or 24 hours. Cells were washed with cold PBS and then lysed with an extraction buffer (50% LC-MS-grade methanol, 30% LC-MS-grade ccetonitrile, 20% ultrapure water). Successively, lysates were incubated for 15 minutes on dry ice and methanol, 15 minutes at 4°C with agitation, and 1 hour at -20°C. Last, samples were centrifuged twice at 16,000 g to remove insoluble debris and the supernatant was analysed by LC-MS/MS.

### LC-MS/MS analysis in cell lines

LC-MS/MS analysis was performed using a Q Exactive Quadrupole-Orbitrap mass spectrometer coupled to a Vanquish UHPLC system (Thermo Fisher Scientific). The liquid chromatography system was fitted with a Sequant ZIC-pHILIC column (150 mm × 2.1 mm) and guard column (20 mm × 2.1 mm) from Merck Millipore and temperature was maintained at 35°C. The sample (2μL) was separated at a flow rate of 0.1 mL/minute. The mobile phase was composed of 10 mM ammonium carbonate and 0.15% ammonium hydroxide in water (solvent A) and acetonitrile (solvent B). A linear gradient was applied by increasing the concentration of solvent A from 20 to 80% within 22 minutes and then maintained for 7 minutes. The mass spectrometer was operated in full MS and polarity switching mode, in the range of 70-1000m/z and resolution 70000. Major ESI source settings were: spray voltage 3.5 kv, capillary temperature 275°C, sheath gas 35, auxiliary gas 5, AGC target 3e6, and maximum injection time 200 minutes. For the targeted analysis, the acquired spectra were analyzed using XCalibur Qual Browser and XCalibur Quan Browser software (Thermo Scientific). The compound discoverer 3.1 (CD) (Thermo Scientific)) was used for untargeted and novel feature detection and annotation with library scoring. Features with the fold change >2 and p <0.05 were selected as discriminating markers. Samples were analysed by quintuplicate.

### Primary samples preparation for UHPLC-MS

CD34^+^ HSPC cells transduced with shSCR or shCOASY were cultured for 10 days in erythroid differentiation medium. Cells were counted, pelleted by centrifugation, and stored at -80°C until analysis. Prior to UHPLC-MS analysis, samples were placed on ice and re-suspended with methanol:acetonitrile:water (5:3:2, v:v) at a concentration of 2 million cells per ml. Suspensions were vortexed continuously for 30 min at 4°C. Insoluble material was removed by centrifugation at 10,000 g for 10 min at 4°C and supernatants were isolated and dried down by vacuum concentration (Labconco). Dried extracts were resuspended in an equal volume of 0.1% formic acid immediately prior to metabolomics analysis by UHPLC-MS.

### UHPLC-MS analysis in primary cells

Analyses were performed as previously published (57, 58). Briefly, the analytical platform employs a Vanquish UHPLC system (Thermo Fisher Scientific) coupled online to a Q Exactive mass spectrometer (Thermo Fisher Scientific). Samples were resolved over a Kinetex C18 column, 2.1 x 150 mm, 1.7 μm particle size (Phenomenex) equipped with a guard column (SecurityGuardTM Ultracartridge – UHPLC C18 for 2.1 mm ID Columns, Phenomenex) using an aqueous phase (A) of water and 0.1% formic acid and a mobile phase (B) of acetonitrile and 0.1% formic acid for positive ion polarity mode, and an aqueous phase (A) of water:acetonitrile (95:5) with 1 mM ammonium acetate and a mobile phase (B) of acetonitrile:water (95:5) with 1 mM ammonium acetate for negative ion polarity mode. Samples were eluted from the column using either an isocratic elution of 5% B flowed at 250 μl/min and 25°C or a gradient from 5% to 95% B over 1 minute, followed by an isocratic hold at 95% B for 2 minutes, flowed at 400 μl/min and 45°C. The Q Exactive mass spectrometer (Thermo Fisher Scientific) was operated independently in positive or negative ion mode, scanning in Full MS mode (2 μscans) from 60 to 900 m/z at 70,000 resolution, with 4 kV spray voltage, 45 sheath gas, 15 auxiliary gas. Calibration was performed prior to analysis using the Pierce Positive and Negative Ion Calibration Solutions (Thermo Fisher Scientific). Acquired data was then converted from .raw to .mzXML file format using Mass Matrix. Samples were analyzed in randomized order with a technical mixture injected after every 10 samples to qualify instrument performance. Metabolite assignments, isotopologue distributions, and correction for expected natural abundances of deuterium, 13C, and 15N isotopes were performed using MAVEN (Princeton).

Volcano plots were prepared using the MetaboAnalyst 5.0 package (www.metaboanalyst.com). Prior to analysis, data were scaled by mean-centering and dividing by the standard deviation of each variable.

### Heme quantification

Heme quantification was performed at day15 after the erythroid differentiation. 100,000 to 200,000 cells were harvested for heme quantification, as previously described (59). Cells were counted, washed with PBS, and then lysed with 100uL formic acid (Sigma). Assay standards with 100ng, 200ng, 400ng, or 800ng of hemin (Sigma) were made for absolute quantification in the same volume of formic acid. After 15 minutes of incubation at room temperature, absorbance was read at 400nM to assess heme content of the samples.

### Coenzyme A dosage assay

5 million of cells per condition were washed in ice cold PBS prior the assay. Cells were then lysed in the CoA assay buffer according to manufacturer’s instructions (Sigma). Lysates were passed 10 times through a 25G syringe and spun down at 10,000g for 10min. Samples were then deproteinised by using 10 KDa spin columns (Abcam). 40uL of the lysate were then used for colorimetric dosage of the Coenzyme A according to manufacturer’s instructions. The final absorbance was measured at 570 nm with the FLUOstar Omega plate reader (BMG Labtech).

### Western Blots

Total cell lysates were obtained by incubating cells in lysis buffer (1% Igepal, CA-630, 0.1% SDS, 0.5% sodium deoxycholate, 100mM NaCl, 50mM Tris-HCL, pH 7.4) supplemented with proteases and phosphatases inhibitors (ThermoFisher). Lysates were then sonicated (30 seconds ON, 30 seconds OFF, 20 cycles, 4°C) with bioruptor Pico sonicator (Diagenode). Cellular debris were eliminated by centrifugation at 16,000 g for 10 minutes at 4°C. Total protein concentration was determined by using a Bicinchoninic Acid Assay kit (ThermoFisher). Loading buffer (ThermoFisher) was then added to each sample and the samples were boiled at 90°C for 5 minutes. Protein lysates were fractionated on 4-12% Bis-Tris Protein Gels (ThermoFisher). Antibodies used for Western blot were: anti-COASY, RRID:AB_11142015; anti-PHGDH, RRID:AB_2737030, and anti-actin, RRID:AB_476693.

### RT-PCR/qPCR and visualisation

RNA was extracted using RNeasy Mini Kit from Qiagen and retrotranscribed with High-Capacity cDNA Reverse Transcription Kit (ThermoFisher). Analysis of *COASY* transcripts by PCR was performed by amplifying *COASY* 5’UTR and all exon-exon junction by using OneTaq Hot Start 2X (NEB). For quantitative PCR analysis, reactions were performed with PowerUp SYBR Green (ThermoFisher). All primers used for PCR and qPCR are in table S2. Separation and visualisation of PCR products was performed on 1% agarose gel or by using Agilent 4200 TapeStation System.

### COASY 5’UTR cloning in dual luciferase lentivector

COASY 5’UTR from NM_001042529.3, NM_001042532.4, NM_025233.7 and XM_011525300.2 transcripts were cloned in the pDualLuc lentivector by VectorBuilder upon request. Forward Primer 5’-GCGGCGGGATCCGTTAGTGCTTCCGGGTTGC-3’, and reverse primer 5’-GCGGCGTCTAGACGGCTGCAGGTGAACATAG-3’ were used for 5’UTR amplification. Purified PCR products were then digested with BamHI and XbaI restriction enzymes and inserted into pDualLuc lentivirus. Sequences were confirmed by DNA sequencing.

### Dual luciferase assay

HEK293T cells transduced with pDualLuc lentiviruses containing the different 5’UTR were used for the dual luciferase assay. 20,000 cells were washed with PBS and the quantification of Luciferase Renilla and Luciferase Firefly activities was achieved by using the Dual-Luciferase Reporter Assay (Promega) according to the manufacturer’s instructions.

### Statistical analysis

Prism Version 8 software (GraphPad) was used for statistical analysis. Data are displayed as the mean±s.d. for cell lines and mean±s.e.m. for primary cells. Statistical analysis was performed using a two-tailed Student’s t-test for comparison of two groups. The Mann Whitney test was specifically used for comparison of primary samples for COASY isoform quantification. A paired two-tailed Student’s t-test was performed for the Coenzyme A dosage comparison. *P* < 0.05 was considered significant. BioRender was used to generate some of the figures, agreement number LG24FKSZ01.

## Supplementary Material

Data file S1

Data file S2

Supplementary Material

## Data Availability

All data associated with this study are present in the paper or Supplementary materials. RNA sequencing FASTQ files are available on the GEO database under the accession number GSE173108. Additional raw data including FCS files and agarose gel pictures are included in the supplementary data. All generated research materials are available upon request by contacting the corresponding author and completion of a materials transfer agreement.

## One Sentence summary

Vitamin B5 and succinyl-CoA compensate *COASY* aberrant splicing in *SF3B1^mut^* myelodysplasia, rescuing heme depletion and ineffective erythropoiesis.
