# Supplementary material for: Vitamin B5 and succinyl-CoA improve ineffective erythropoiesis in SF3B1 mutated myelodysplasia
Source: Sci Transl Med. Author manuscript; Available in PMC 2023 May 10. (PMC7614516; doi:10.1126/scitranslmed.abn5135)
Supplement: Supplementary Material [file EMS175159-supplement-Supplementary_Material.pdf]

Science Translational Medicine
AAAS

Supplementary Materials for

Vitamin B5 and succinyl-CoA improve ineffective erythropoiesis in *SF3B1*-mutated myelodysplasia

Syed A. Mian *et al.*

Corresponding author: Kevin Rouault-Pierre, k.rouault-pierre@qmul.ac.uk

Sci. Transl. Med. **15**, eabn5135 (2023)
DOI: 10.1126/scitranslmed.abn5135

The PDF file includes:

Figs. S1 to S7
Tables S1 and S2
Legends for data files S1 and S2

Other Supplementary Material for this manuscript includes the following:

Data files S1 and S2
MDAR Reproducibility Checklist

Supplementary Figures:

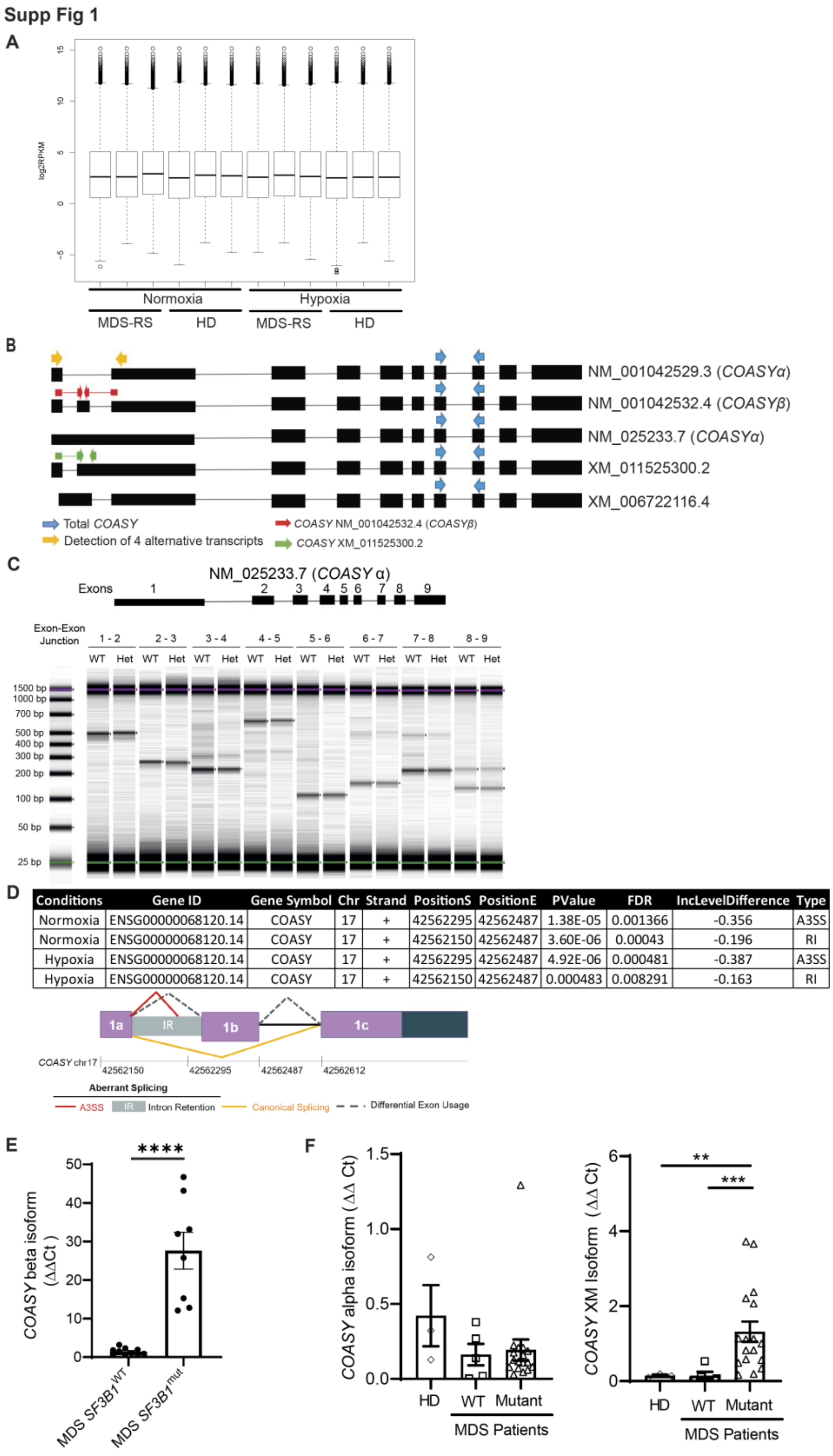

Conditions	Gene ID	Gene Symbol	Chr	Strand	PositionS	PositionE	PValue	FDR	IncLevelDifference	Type
Normoxia	ENSG00000068120.14	COASY	17	+	42562295	42562487	1.38E-05	0.001366	-0.356	A3SS
Normoxia	ENSG00000068120.14	COASY	17	+	42562150	42562487	3.60E-06	0.00043	-0.196	RI
Hypoxia	ENSG00000068120.14	COASY	17	+	42562295	42562487	4.92E-06	0.000481	-0.387	A3SS
Hypoxia	ENSG00000068120.14	COASY	17	+	42562150	42562487	0.000483	0.008291	-0.163	RI

Figure S1: Splicing profiling of MDS-RS patient samples reveals alternative splicing of the coenzyme A synthase (*COASY*).

(A) Boxplot with log2(RPKM) showing the RNA sequencing data quality for MDS-RS *SF3B1*^{mut} and healthy donor (HD) samples. **(B)** Representation of primers designed for *COASY* transcripts used for qPCR analysis. **(C)** Representative gel image obtained by tape station analysis of *COASY* RT-PCR in K562 *SF3B1* wild type (WT) and *SF3B1* heterozygous mutant (Het). Primers were designed to amplify every exon-exon junctions in *COASY* transcript. **(D)** Upper Panel: Table representing the differential splicing analyses of COASY events characterised by rMATS in the primary patient samples under normoxia and hypoxia conditions. A3SS = Alternative 3' Splice Site; RI = Retained Intron; FDR = False Discovery Rate. Lower panel: Schematic representing the canonical splicing site and differential exon usage of COASY in *SF3B1* mutants. The alternative 3' splice site (A3SS) event is represented in red and the retained intron (RI) by a grey box. Thus the differential exon usage leads to the inclusion of exon 1b in the isoform coding for COASY β. **(E)** Quantitative PCR analysis, on a validation cohort, of NM_001042532.4 isoform encoding *COASY* β in CD34^{+} from healthy donors (n=3), MDS *SF3B1*^{WT} (n=10) and patients with MDS *SF3B1*^{mut} (n=8). **(F)** Quantitative PCR analysis of NM_001042529.3 isoform encoding COASY α (left panel) and XM_011525300.2 (right panel) in healthy donors (n=3) and patients with MDS *SF3B1*^{WT} (n=5) or MDS *SF3B1*^{mut} (n=17). Data presented here are the mean± S.E.M. ** p<0.01, *** p<0.001.

Supp Fig 2

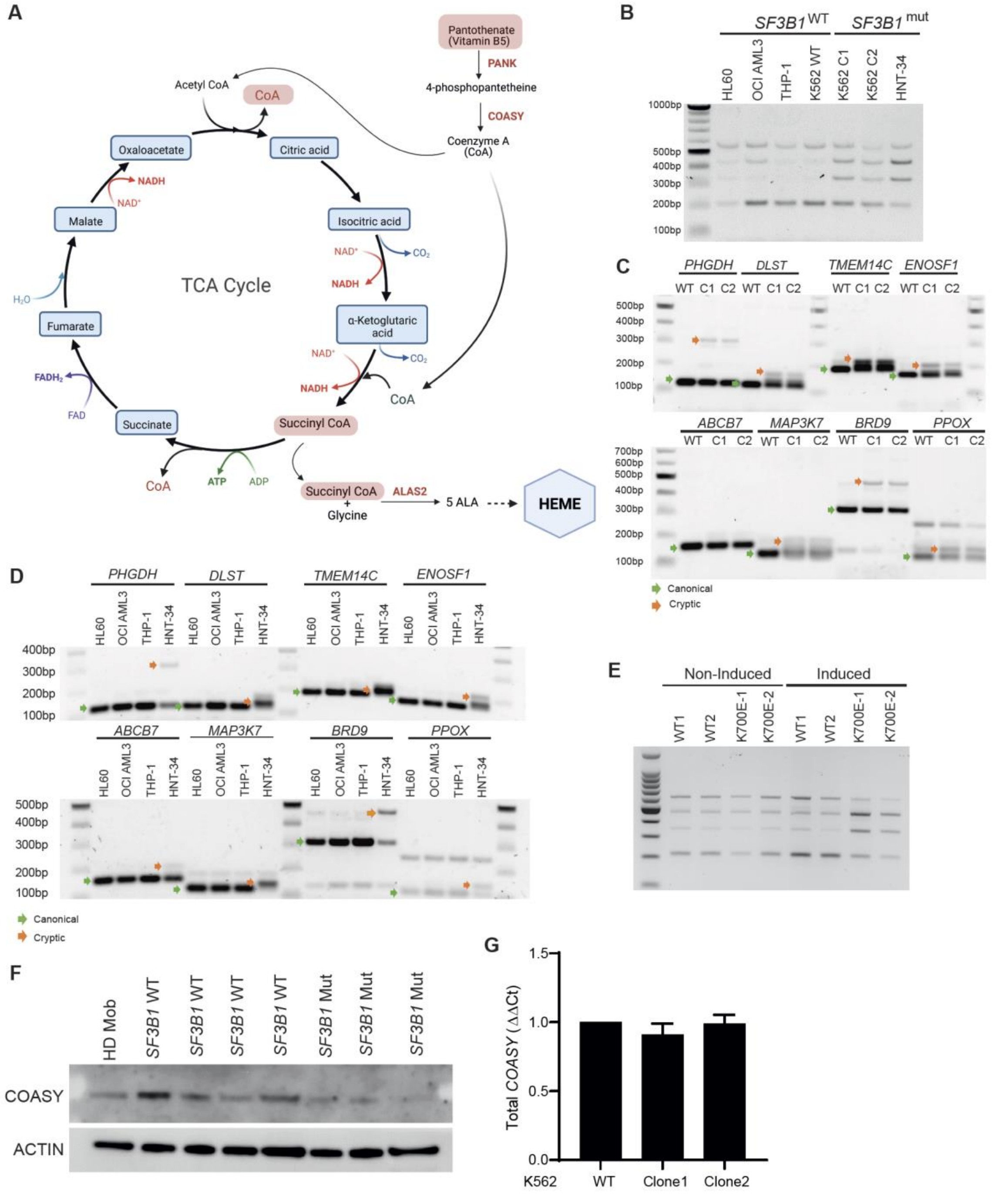

Figure S2: *COASY* mis-splicing and known mis-splicing events are replicated in the *SF3B1* mutated cell lines.

(A) Schematic of CoA biosynthesis and its integration into the TCA cycle. Succinyl-CoA, an intermediate metabolite of the TCA cycle, condenses with glycine through a reaction catalyzed by ALAS2 to initiate heme synthesis. **(B)** Representative DNA gel image of the *COASY* 5'UTR RT-PCR in *SF3B1*^{wt} cell lines (HL60, OCI AML3, THP-1, K562 WT) and *SF3B1*^{mut} cell lines (K562 Clone1,

Clone2 and HNT-34). Isoforms amplified are NM_025233.7 (COASY α) at 604bp; XM_011525300.2 at 468bp; NM_001042532.4 (COASY β) at 344bp, NM_001042529.3 (COASY α) at 212bp. **(C)** Representative gel image of reported mis-splicing events in genes (*PHGDH*, *DLST*, *TMEM14C*, *ENOSF1*, *ABCB7*, *MAP3K7*, *BRD9* and *PPOX*) analysed by RT-PCR in K562 *SF3B1*^{WT} and K562 *SF3B1*^{mut} cells (Clone C1 and C2). **(D)** Representative gel image of reported mis-splicing events in genes (*PHGDH*, *DLST*, *TMEM14C*, *ENOSF1*, *ABCB7*, *MAP3K7*, *BRD9* and *PPOX*) analysed by RT-PCR in *SF3B1*^{wt} cell lines (OCI AML3 and THP-1) and *SF3B1*^{mut} cell lines (HNT-34). **(E)** Representative gel image of the *COASY* 5'UTR RT-PCR in *SF3B1*^{WT} (WT1, WT2) or Dox inducible *SF3B1*^{mut} K562 (K700E-1, K700E-2). Isoforms amplified are NM_025233.7 (COASY α) at 604bp; XM_011525300.2 at 468bp; NM_001042532.4 (COASY β) at 344bp, NM_001042529.3 (COASY α) at 212bp. **(F)** Western blot showing COASY protein expression in 1 $CD34^+$ mobilised from a healthy donor (HD mob), n=4 *SF3B1*^{WT} sampoles, and n=3 *SF3B1*^{mut} patient samples. Actin B protein was used as a loading control. **(G)** Quantitative PCR analysis of total *COASY* mRNA expression in K562 cell line *SF3B1*^{WT} and *SF3B1*^{mut} (Clone 1&2) (n=3). Data presented here are the mean± S.D.

Supp Fig 3

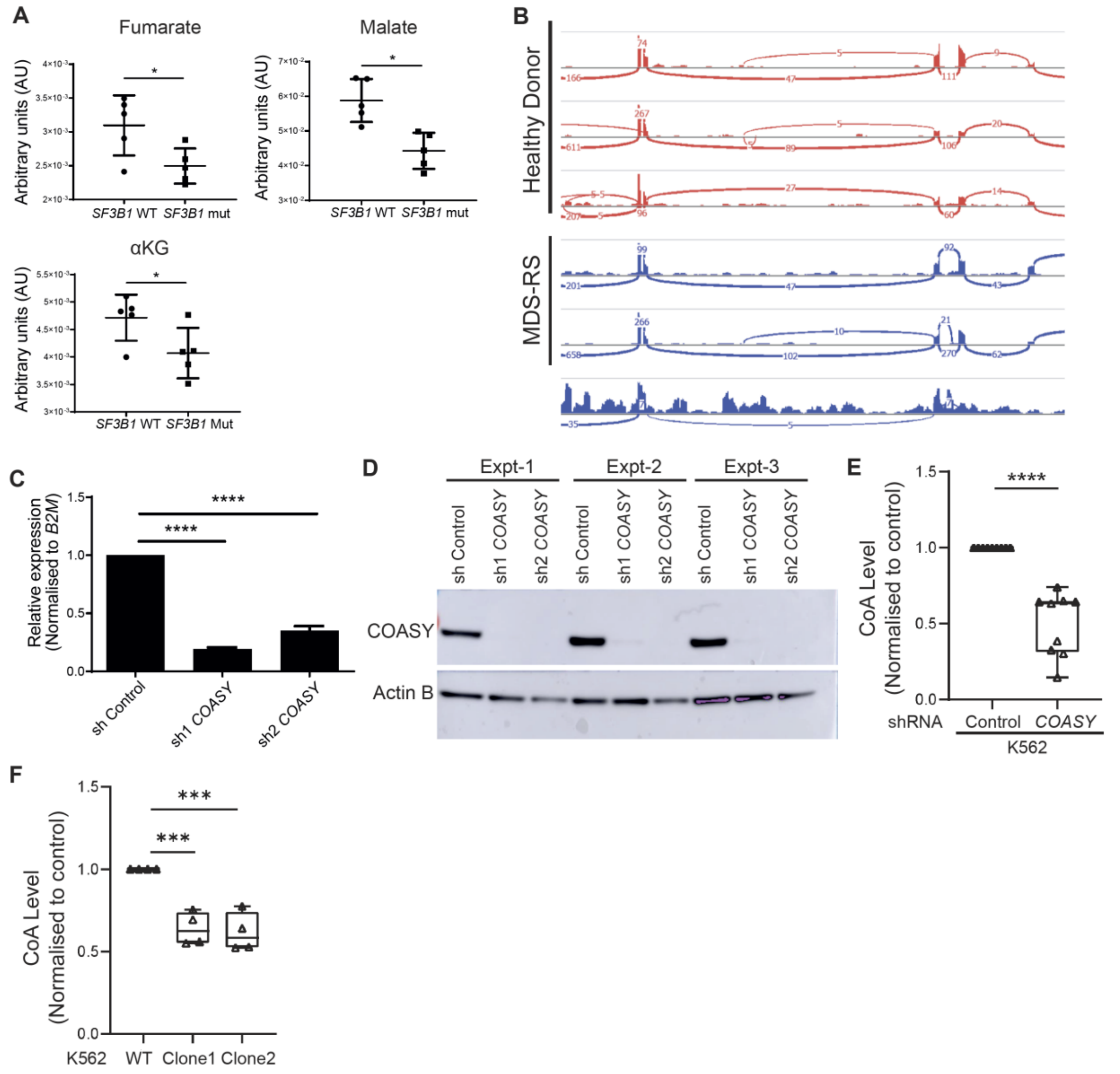

Figure S3: Metabolites are depleted in TCA cycle of *SF3B1* mutant cells, *PHGDH* is mis-spliced in primary patient samples, and validation of *COASY*'s knock down efficiency.

(A) LC-MS/MS analysis showing the concentrations of fumarate, malate and α-ketoglutarate in K562 *SF3B1*^{wt} and K562 *SF3B1*^{mut} (N=5) cells. Data presented here is the mean ± S.E.M * $p<0.05$. **(B)** Sashimi plots from RNA sequencing data representing read coverage in RPKM at exon-exon junction in *PHGDH* transcript (exon 5-6) for 3 patients with MDS *SF3B1*^{mut} (in blue) and 3 healthy donors (in red). Numbers of reads covering the junctions are displayed on the plot. **(C)** Quantitative PCR analysis of total *COASY* expression in K562 transduced with shControl (n=3) or shCOASY1 (n=3) and shCOASY2 (n=3). Data presented here are the mean± S.D **** $p<0.0001$. **(D)** Representative Western

blot showing COASY protein expression in K562 transduced with shControl (n=3) or shCOASY1 (n=3) and shCOASY2 (n=3). Actin B protein is used as a loading control. **(E)** Quantification of CoA concentrations in K562 cell line with either shControl or shCOASY (n=3). Data presented here are the mean± S.D **** $p<0.0001$. **(F)** Quantification of CoA concentrations in K562 cell line with either *SF3B1*^{wt} or *SF3B1*^{mut} (Clone 1&2) (n=4). Data presented here are the mean± S.D *** $p<0.001$.

Supp Fig 4

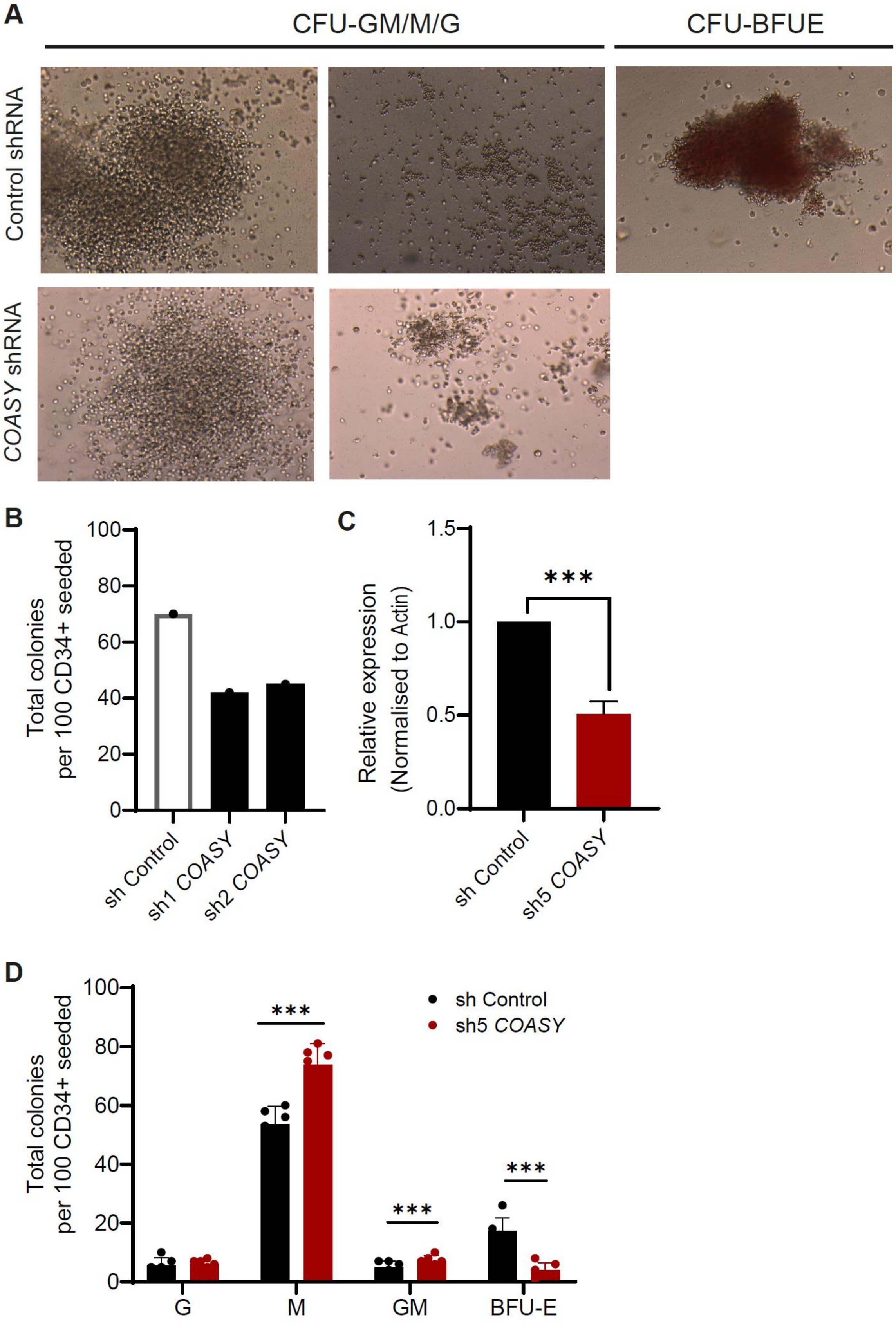

A
CFU-GM/M/G
CFU-BFUE
Control shRNA
COASY shRNA
B
Total colonies per 100 CD34+ seeded
0
20
40
60
80
100
sh Control
sh1 COASY
sh2 COASY
C
Relative expression (Normalised to Actin)
0.0
0.5
1.0
1.5
***
sh Control
sh5 COASY
D
Total colonies per 100 CD34+ seeded
0
20
40
60
80
100
sh Control
sh5 COASY
***
***
***
G
M
GM
BFU-E

Figure S4: *COASY* knock down impairs erythroid differentiation of human primary hematopoietic cells.

(A) Representative pictures of colonies (CFU-GM/M/G and CFU/BFU-E) obtained with shCOASY and shControl transduced $CD34^+$ HSPCs cells seeded in methylcellulose, at day14. **(B)** Total colonies per 100 $CD34^+$ cells transduced with shControl or shCOASY1 or 2, and seeded in methycellulose for 14 days (n=1). **(C)** Quantitative PCR analysis of COASY total expression. shRNA#5 directed against *COASY* showed a reduction of expression of approximately 50%, which reproduced similar expression to that observed in patient samples. **(D)** Colonies per 100 $CD34^+$ cells transduced with shControl or shCOASY5, and seeded in methycellulose for 14 days (n=1). *BFU-E, Burst forming unit erythroid; G, Granulocyte; M, Monocytes; GM, Granulocyte-macrophage.*

Supp Fig 5

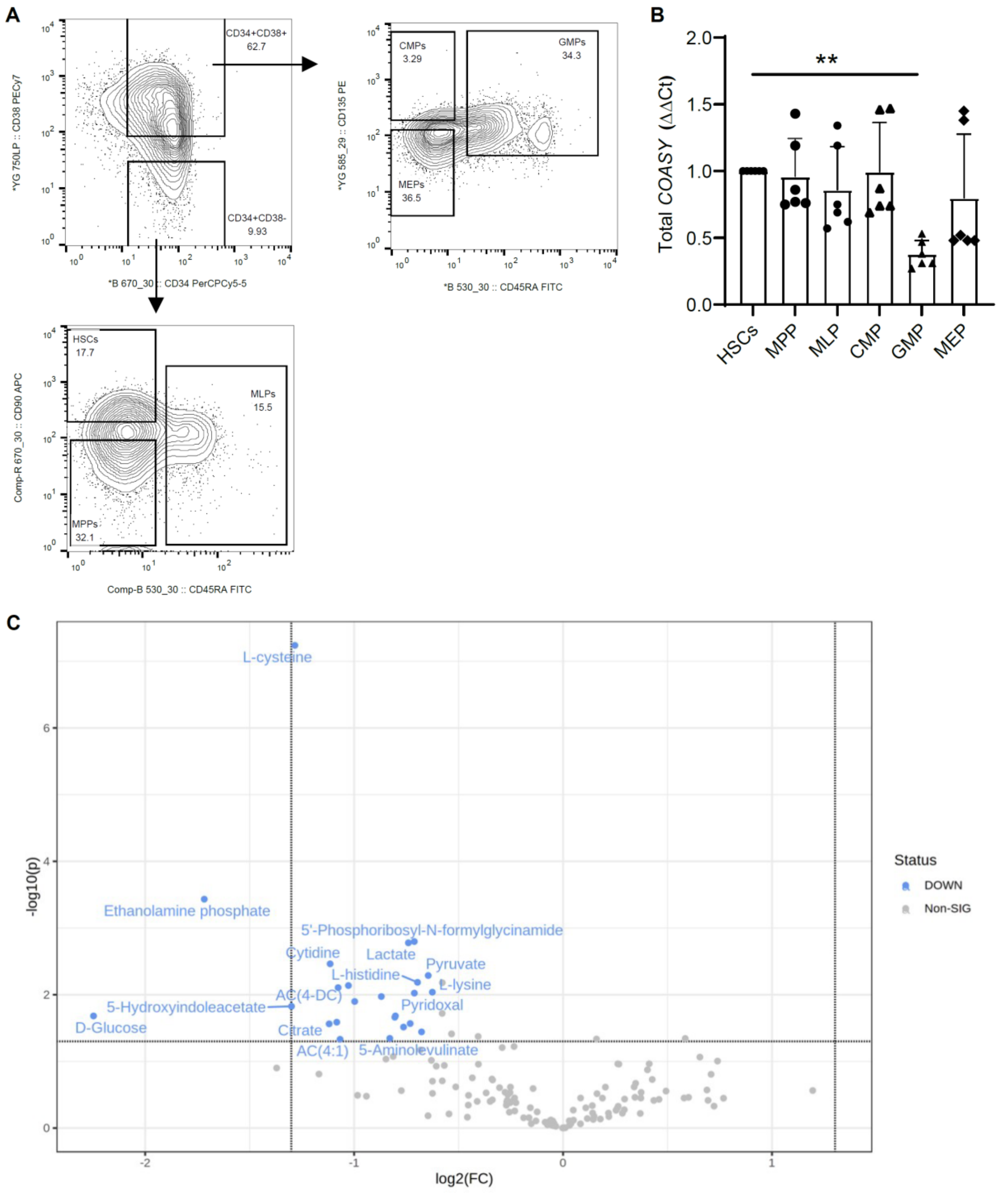

Figure S5: COASY is expressed throughout the differentiation hierarchy of haematopoietic stem and progenitor cells.

(A) Schematic representation of cell fractions (haematopoietic stem cells, HSCs; Multipotent progenitor cells, MPPs; Multi-lymphoid progenitors, MLPs; Common myeloid progenitor, CMPs; Granulocyte/monocyte progenitor, GMPs; Megakaryocyte–erythroid progenitors, MEPs) isolated from

cord blood mononuclear cells using FACS. **(B)** Quantitative PCR analysis of total *COASY* expression in HSCs, MPPs, MLPs, CMPs, GMPs. Three independent cord blood donor pools were used for this analysis. Data presented here are the mean± S.E.M ** $p<0.01$. **(C)** Volcano plot, generated from UHPLC-MS analysis, depicts the differential metabolites (*pvalue*< 0.05) identified between $CD34^+$ HSPCs transduced with shControl and $CD34^+$ HSPCs transduced with shCOASY undergoing erythroid differentiation for 10 days.

Supp Fig 6

A

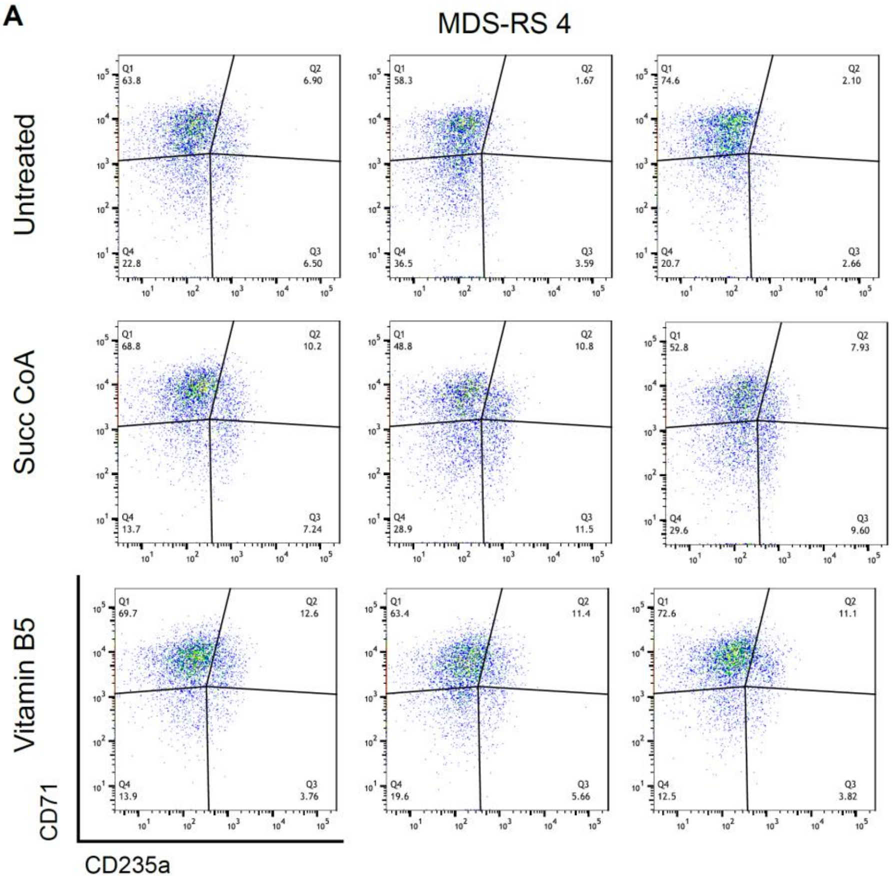

MDS-RS 4
Untreated
Succ CoA
Vitamin B5
CD71
CD235a

B

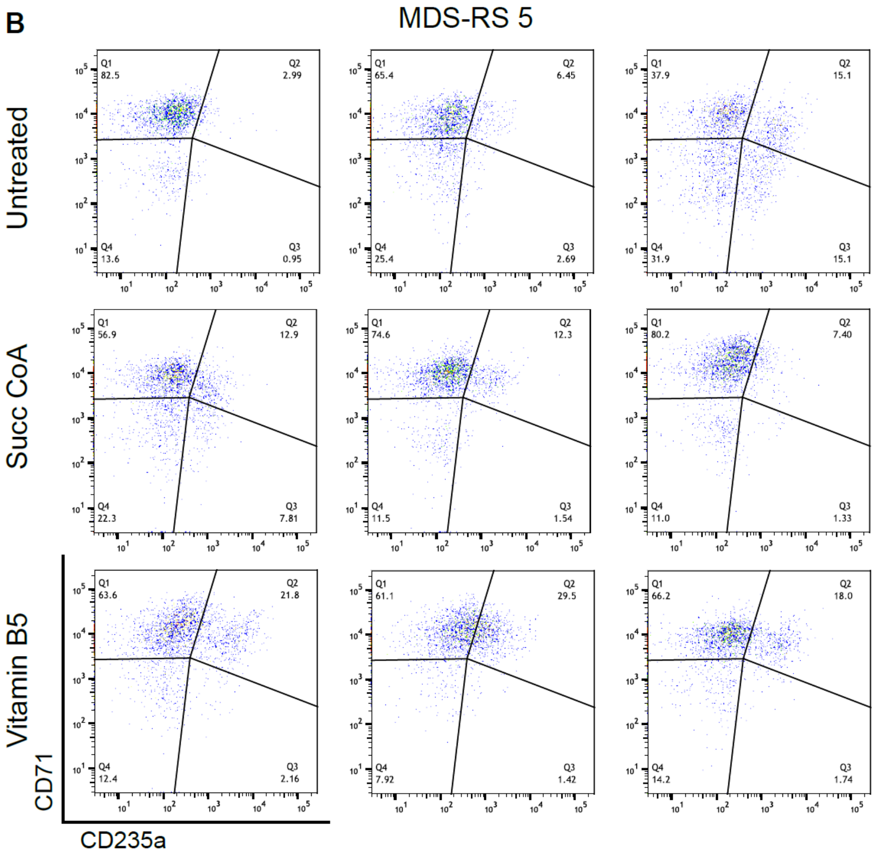

MDS-RS 5
Untreated
Succ CoA
Vitamin B5
CD71
CD235a

Supp Fig 6

C

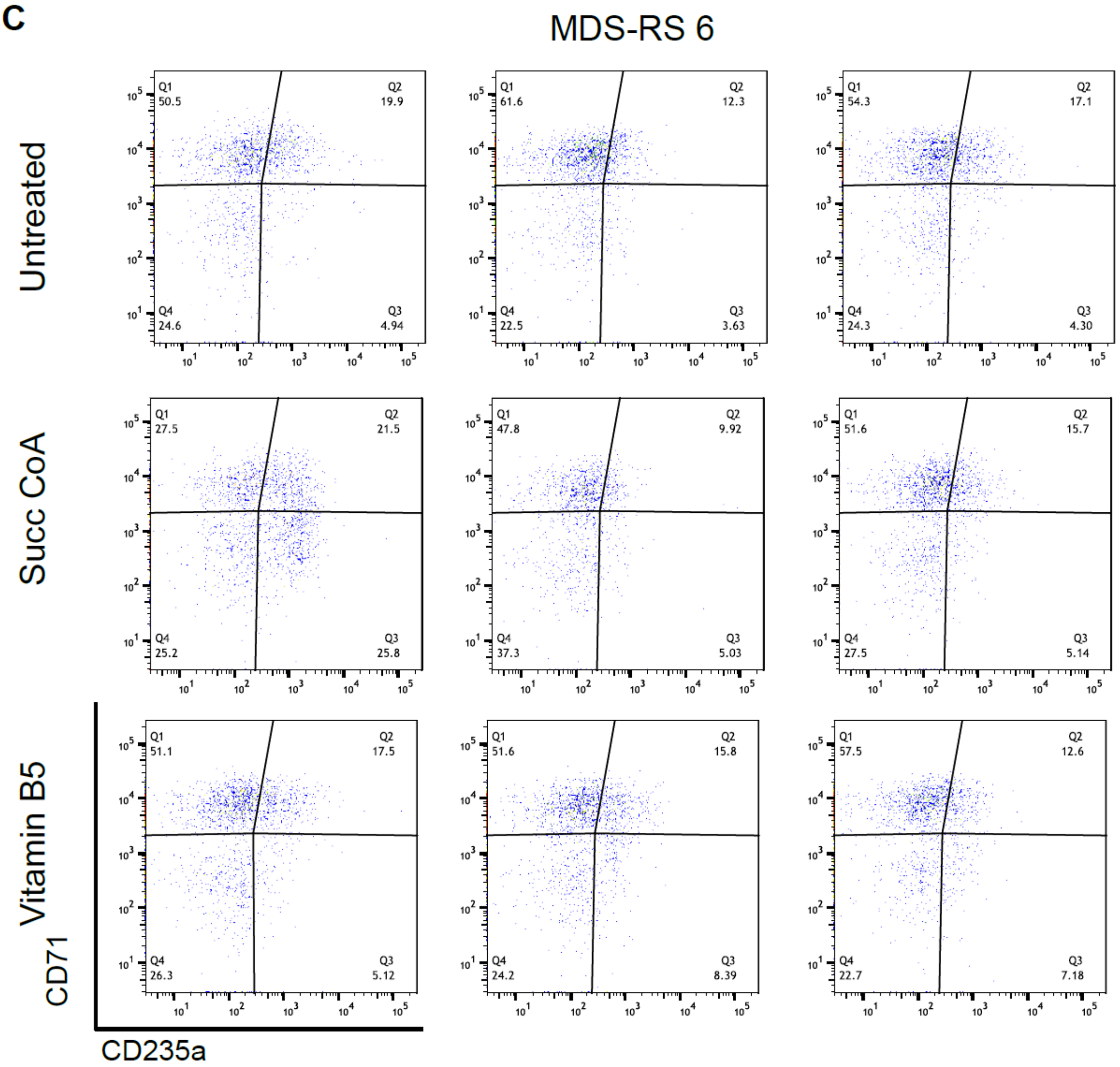

MDS-RS 6
Untreated
Succ CoA
Vitamin B5
CD71
CD235a
Q1
50.5
Q2
19.9
Q4
24.6
Q3
4.94
Q1
61.6
Q2
12.3
Q4
22.5
Q3
3.63
Q1
54.3
Q2
17.1
Q4
24.3
Q3
4.30
Q1
27.5
Q2
21.5
Q4
25.2
Q3
25.8
Q1
47.8
Q2
9.92
Q4
37.3
Q3
5.03
Q1
51.6
Q2
15.7
Q4
27.5
Q3
5.14
Q1
51.1
Q2
17.5
Q4
26.3
Q3
5.12
Q1
51.6
Q2
15.8
Q4
24.2
Q3
8.39
Q1
57.5
Q2
12.6
Q4
22.7
Q3
7.18

Supp Fig 6

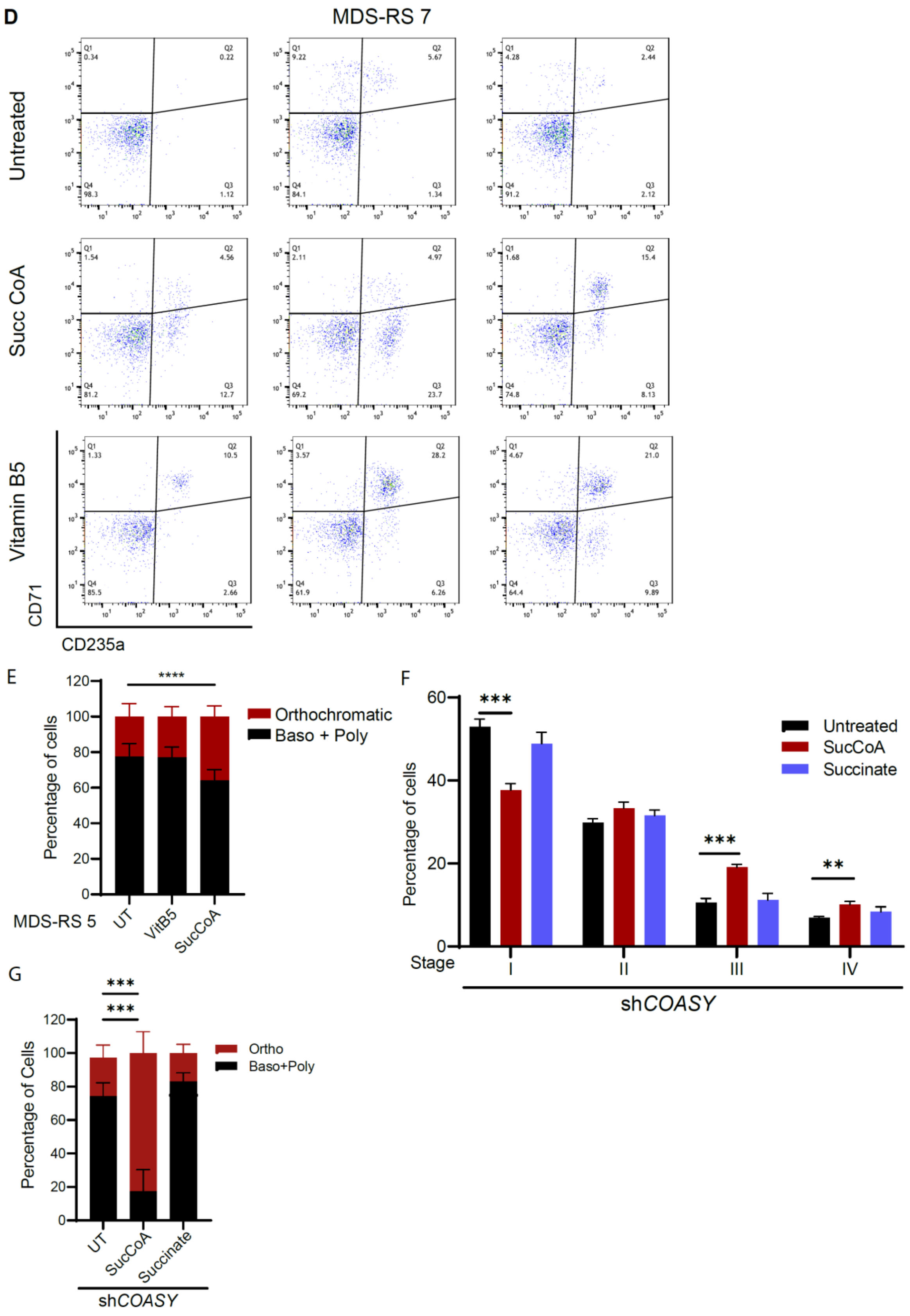

Figure S6: Vitamin B5 and Succinyl-CoA rescue erythroid differentiation defect observed in MDS-RS *SF3B1*^{mut}

(A-D) Flow cytometry analysis of CD71/CD235a acquisition in $CD34^+$ cells from patients with MDS *SF3B1*^{mut}, treated with/without vitamin B5 or succinyl-CoA, at day 14 of erythroid differentiation. Dot plots represent experiment carried out on 4 independent patient samples (MDS-RS 4(A)/ 5(B)/ 6(C)/ 7(D)) in triplicate. **(E)** Quantification of erythroid cell based on the cellular morphology using Giemsa staining of $CD34^+$ MDS-RS cells (patient MDS-RS5) treated with/without succinyl-CoA or additional vitamin B5 following erythroid differentiation for 14 days. Basophilic/polychromatic and orthochromatic erythoblasts were counted from 10 representative fields for each condition (n=3). Data presented here are the mean ±S.E.M. **** $p<0.001$. **(F)** Flow cytometry analysis of CD71/CD235a acquisition from $CD34^+$ UCB cells undergoing erythroid differentiation for 14 days, and treated with/without succinyl-CoA or succinate at day14. Percentage of cells in quadrants I ($CD71^-/CD235a^-$), II ($CD71^+/CD235a^-$), III ($CD71^+/CD235a^+$) and IV ($CD71^-/CD235a^+$) are reported (n=3). Data presented here are the mean± S.E.M ** $p<0.01$, *** $p<0.005$. **(G)** Quantification of erythroid cell morphology using Giemsa staining of shCOASY $CD34^+$ HSPCs cells undergoing erythroid differentiation for 14 days, and treated with/without succinyl-CoA or succinate. basophilic/polychromatic and orthochromatic erythroblasts were counted from 10 representative fields for each replicate (n=3). Data presented here are the mean ±S.E.M. *** $p<0.005$.

Supp Fig 7

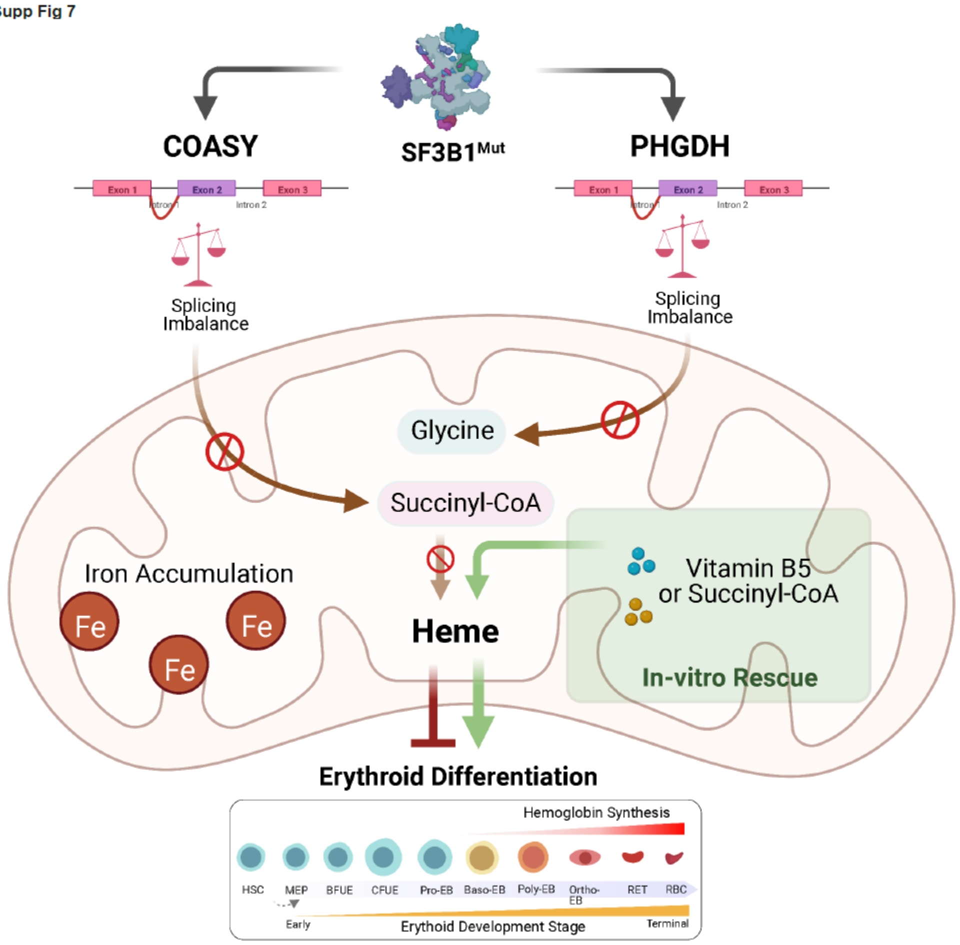

Figure S7: *COASY* and *PHGDH* mis-splicing contribute to the heme synthesis defect and erythroid differentiation blockage observed in MDS-RS *SF3B1*^{mut}.

Graphical representation showing that *SF3B1* mutation causes mis-splicing in *COASY* and *PHGDH* transcripts and results in the loss of their respective protein. Deficiency in these two enzymes depletes cells in succinyl-CoA and glycine which are the two metabolites involved in the rate limiting step of heme synthesis. Treatment of MDS *SF3B1* mutant cells with vitamin B5 (precursor of CoA), or succinyl-CoA, rescues erythroid differentiation in these cells.

Supplementary Tables

Table S1: Patient clinical data.

#	MDS WHO	SF3B1 mut	SF3B1 mut variant	VAF	Experiment
1	MDS-RS-SLD	yes	H662Q	40%	RNAseq/qPCR/PCR
2	MDS-RS-MLD	yes	K700E	50%	RNAseq/qPCR/PCR
3	MDS-RS-MLD	yes	H662Q	47%	RNAseq/qPCR/PCR
4	MDS-RS-MLD	yes	K700E	37.90%	Erythroid differentiation in vitro
5	MDS-RS-MLD	yes	K700E	49%	Erythroid differentiation in vitro
6		yes	H662Q	41.20%	Erythroid differentiation in vitro
7		yes			Erythroid differentiation in vitro
8		yes			qPCR
9		yes			qPCR
10	MDS-RS-MLD	yes			qPCR
11	MDS-RS-SLD	yes			qPCR
12	MDS-RS-MLD	yes			qPCR
13	MDS-RS-SLD	yes			qPCR
14	MDS-RS-SLD	yes			qPCR
15		yes			qPCR
16	MDS-RS-MLD	yes			qPCR
17	MDS-RS-MLD	yes			qPCR
18	MDS-EB1	yes			qPCR
19	MDS-EB1	yes			qPCR
20	MDS/MPN	yes			qPCR
21	MDS-RS-MLD	yes			qPCR
22	MDS-RS-SLD	yes	R625C	33%	qPCR
23	MDS-RSMDS/MPN-RS- with Thrombocytosis	yes	K700E	48%	qPCR
24	MDS-RS-SLD	yes	E622D	40%	qPCR
25	MDS-RS-MLD	yes	R625C	36.50%	qPCR
26	MDS-RS-SLD	yes	K700E	37%	qPCR
27	MDS-RS-SLD	yes	K666N	39%	qPCR
28	MDS-RS-SLD	yes	K666N	12.30%	qPCR
29	MDS-RS-MLD	yes	E622D	41%	qPCR
30	MDS-MLD	no			RNAseq/qPCR/PCR
31	MDS-MLD	no			RNAseq/qPCR/PCR
32	MDS-EB	no			RNAseq/qPCR/PCR
33	MDS-EB1	no			qPCR
34	MDS-MLD	no			qPCR
35	ICUS	no			qPCR
36	ICUS	no			qPCR
37	ICUS	no			qPCR
38	ICUS	no			qPCR
39	MDS-SLD	no			qPCR
40	MDS-MLD	no			qPCR
41	MDS-MLD	no			qPCR
42	MDS-MLD	no			qPCR
43	MDS-MLD	no			qPCR
44	MDS-MLD	no			qPCR
45	MDS-RS-MLD	no			qPCR
46	MDS-EB1	no			qPCR

Table S2: Primers

Gene	Sequence
ABCB7 FW	5'-ATGATGCAGGTAATGCTGCT-3'
ABCB7 RV	5'-TCAGCATAGCCAGAGTAGAGGT-3'
ACTB FW	5'-GCCGCCAGCTCACCAT-3’
ACTB RV	5’-TCGTCGCCCACATAGGAATC-3’
B2M FW	5’-AGCAGCATCATGGAGGTTTGA-3’
B2M RV	5’-TCAAACATGGAGACAGCACTCA-3’
BRD9 FW	5'-AGCTCTGTTCTGGAGTTCATG-3'
BRD9 RV	5'-CTGAAGAAACTCATAGGGGTCGTG-3'
COASY 5'UTR FW	5'-CGGCTGCAGGTGAACATAG-3'
COASY 5'UTR RV	5'-GTTAGTGCTTCCGGGTTGC-3'
COASY All variants (Total) FW	5'-ATGACAGCAGTCCACACCTC-3'
COASY All variants (Total) RV	5'-CCAATTATCGCAAAGCTGGC-3'
COASY exon 1-2 FW	5’-CCTCGGTGCTGCTATACTCC-3’
COASY exon 1-2 RV	5’-CGATAGGTCTCCTCGCTGAC-3’
COASY exon 2-3 FW	5’-CTTTTGATGTCATCCCCCTGC-3’
COASY exon 2-3 RV	5’-GTTCCCCAACATTCGCTGG-3’
COASY exon 3-4 FW	5’-AGGAACTTGCTTTGTACCAGA-3’
COASY exon 3-4 RV	5’-AGCTATTGAGCTCTTCCCAGA-3’
COASY exon 4-5 FW	5’-ATTGACAGTGACCACCTGGG-3’
COASY exon 4-5 RV	5’-TGCCTAGGACCTTCCTGTTG-3’
COASY exon 5-6 FW	5’-GGCATCATCAACAGGAAGGTC-3’
COASY exon 5-6 RV	5’-CCAGCTTTGCGATAATTGGC-3’
COASY exon 6-7 FW	5’-CCAATTATCGCAAAGCTGGC-3’
COASY exon 6-7 RV	5’-ATGACAGCAGTCCACACCTC-3’
COASY exon 7-8 FW	5’-TGTGTGTGTGATTGATGCCG-3’
COASY exon 7-8 RV	5’-CGTGGCTCTGTTCCACAAG-3’
COASY exon 8-9 FW	5’-CTTGTGGAACAGAGCCACG-3’
COASY exon 8-9 RV	5’-GAGGGCCTGATGAGTCTTGG-3’
COASY NM_001042532.4 (beta) FW	5'-CAAGCCTTGAGGTTTCATTTCCCC-3'
COASY NM_001042532.4 (beta) RV	5'-CTGCCCAGGCCGACAGGA-3'
COASY NM_025233.7 (alpha) FW	5'-CTGTGACAAGGGTTCCTGTCC-3'
COASY NM_025233.7 (alpha) RV	5'-GGGTTTCAGTGCTGACCGAC-3'
COASY XM_011525300.2 FW	5'-CCTTGAGGTTTCATTTCCCC-3'
COASY XM_011525300.2 RV	5'-CCTGTATGGGACCTTGGAC-3'
DLST FW	5'-AGCTGTATGCAAGGATGACTTGG-3'
DLST RV	5'-CATCTTCTGCAACTGTGTCTCC-3'
ENOSF1 FW	5'-GGGTGCTGATCTCCAGGATG-3'
ENOSF1 RV	5'-GGAGGTTGGCTCCTCAATCC-3'
MAP3K7 FW	5'-GATGGAATATGCTGAAGGGG-3'
MAP3K7 RV	5'-CACTCCTTGGGAACACTGTA-3'
PHGDH FW	5'-CCTCCTTTGGTGTTCAGCAGC-3'
PHGDH RV	5'-GGCAAAGGTGTTGTCATTCAGC-3'
PPOX FW	5'-GGCCCTAATGGTGCTATCTTTG-3'
PPOX RV	5'-CTTCTGAATCCAAGCCAAGCTC-3'
TMEM14C FW	5'-GACACCTCGCAGTCATTCCT-3'
TMEM14C RV	5'-TGATCCCACCAGAAGCAACC-3'

Supplementary data files

Data file S1: List of the 200 genes exclusively seen in MDS SF3B1 mutant patient cells.

Data file S2: List of the top 40 mis-splicing events observed in MDS SF3B1 mutant patient cells upon normoxia and hypoxia conditions.
